# Complex organic matter degradation by secondary consumers in chemolithoautotrophy-based subsurface geothermal ecosystems

**DOI:** 10.1371/journal.pone.0281277

**Published:** 2023-08-18

**Authors:** Raegan Paul, Timothy J. Rogers, Kate M. Fullerton, Matteo Selci, Martina Cascone, Murray H. Stokes, Andrew D. Steen, J. Maarten de Moor, Agostina Chiodi, Andri Stefánsson, Sæmundur A. Halldórsson, Carlos J. Ramirez, Gerdhard L. Jessen, Peter H. Barry, Angelina Cordone, Donato Giovannelli, Karen G. Lloyd

**Affiliations:** 1 Microbiology Department, University of Tennessee, Knoxville, TN, United States of America; 2 Department of Biology, University of Naples “Federico II”, Naples, Italy; 3 Observatorio Volcanológico y Sismológico de Costa Rica (OVSICORI) Universidad Nacional, Heredia, Costa Rica; 4 Department of Earth and Planetary Sciences, University of New Mexico, Albuquerque, NM, United States of America; 5 Instituto de Bio y Geociencias del NOA (IBIGEO, UNSa-CONICET), Salta, Argentina; 6 NordVulk, Institute of Earth Sciences, University of Iceland, Reykjavík, Iceland; 7 Servicio Geologico Ambiental, Heredia, Costa Rica; 8 Instituto de Ciencias Marinas y Limnológicas, Universidad Austral de Chile, Valdivia, Chile; 9 Center for Oceanographic Research COPAS COASTAL, Universidad de Concepción, Concepción, Chile; 10 Marine Chemistry & Geochemistry Department, Woods Hole Oceanographic Institution, Woods Hole, MA, United States of America; 11 National Research Council–Institute of Marine Biological Resources and Biotechnologies—CNR-IRBIM, Ancona, Italy; 12 Department of Marine and Coastal Science, Rutgers University, New Brunswick, NJ, United States of America; 13 Earth-Life Science Institute, Tokyo Institute of Technology, Tokyo, Japan; University of Nebraska-Lincoln, UNITED STATES

## Abstract

Microbial communities in terrestrial geothermal systems often contain chemolithoautotrophs with well-characterized distributions and metabolic capabilities. However, the extent to which organic matter produced by these chemolithoautotrophs supports heterotrophs remains largely unknown. Here we compared the abundance and activity of peptidases and carbohydrate active enzymes (CAZymes) that are predicted to be extracellular identified in metagenomic assemblies from 63 springs in the Central American and the Andean convergent margin (Argentinian backarc of the Central Volcanic Zone), as well as the plume-influenced spreading center in Iceland. All assemblies contain two orders of magnitude more peptidases than CAZymes, suggesting that the microorganisms more often use proteins for their carbon and/or nitrogen acquisition instead of complex sugars. The CAZy families in highest abundance are GH23 and CBM50, and the most abundant peptidase families are M23 and C26, all four of which degrade peptidoglycan found in bacterial cells. This implies that the heterotrophic community relies on autochthonous dead cell biomass, rather than allochthonous plant matter, for organic material. Enzymes involved in the degradation of cyanobacterial- and algal-derived compounds are in lower abundance at every site, with volcanic sites having more enzymes degrading cyanobacterial compounds and non-volcanic sites having more enzymes degrading algal compounds. Activity assays showed that many of these enzyme classes are active in these samples. High temperature sites (> 80°C) had similar extracellular carbon-degrading enzymes regardless of their province, suggesting a less well-developed population of secondary consumers at these sites, possibly connected with the limited extent of the subsurface biosphere in these high temperature sites. We conclude that in < 80°C springs, chemolithoautotrophic production supports heterotrophs capable of degrading a wide range of organic compounds that do not vary by geological province, even though the taxonomic and respiratory repertoire of chemolithoautotrophs and heterotrophs differ greatly across these regions.

## Introduction

Terrestrial geothermal systems emit volatiles from the Earth’s interior (i.e., mantle and crust) to the atmosphere. Often, meteoric water permeates into the subsurface hydrothermal system, where it is heated and rises to the surface, bringing with it volatiles from the deep subsurface [1]. The differential enrichment of these volatiles into geothermal fluids creates environmental niches that can be saturated with deeply-derived inorganic carbon and other compounds [2–4]. In convergent margins such as those of the Central American and the Andean Central Volcanic Zone, inorganic carbon is derived from the mantle, overlying crust and/or down-going slab [5]. In divergent spreading centers and/or areas with mantle plume-influenced volcanism, such as Iceland, geothermal systems are often dominated by deep mantle gases (e.g., Harðardóttir et al. 2018 [6]). These geological systems create a large diversity of surface-emitting springs that range in temperature, pH, inorganic carbon content, and availability of redox active compounds that together make the driving force of microbial community composition [4, 7, 8]. Sampling fluid emissions from natural surface springs provides access to these deeply-sourced microbial communities and the volatiles that support them [8–11].

The important role that chemolithoautotrophs play in these geothermal ecosystems is well-established (e.g., [4, 12–14]). The heterotrophic communities within these systems are less often studied, even though heterotrophs have been shown to be dominant within heavily-sedimented subsurface ecosystems (e.g., [15]). These heavily-sedimented systems do not have a constant supply of redox active volatiles and are therefore dependent on allocthonously-derived organic matter, and likely differ greatly from geothermal ecosystems. Recent work has focused on understanding the heterotrophic community within terrestrial geothermal systems [16, 17] and many industrially useful carbohydrate- and peptide-degrading enzymes have been isolated from these microbial communities [18, 19]. Specifically, carbohydrate active enzymes and peptidases have been found in hot spring fluids [17, 20, 21]. However, a survey of the full complement of all the carboyhdrate active enzymes and peptidases have not been made from metagenomes from hot springs, to our knowledge. The taxonomy and respiratory pathways of primary producers and heterotrophs are known to vary along geological gradients according to changes in deep volatile delivery [4, 7, 8]. However, it is not known whether organic carbon degradation pathways vary along with them. Different types of chemolithoautotrophs may promote different compositions of carbon-degrading enzymes in the ecosystems they support. Thus, it is important to investigate the heterotrophic community because it actively participates in the geochemical cycle of terrestrial geothermal environments by consuming organic carbon and releasing inorganic carbon.

Hot springs typically contain little photosynthetically-derived organic matter, potentially leading heterotrophs to depend primarily on the byproducts of the chemolithoautotrophic community [22]. To access these organic byproducts, heterotrophs use extracellular enzymes to break down larger organic molecules into smaller molecules that can permeate their cell membrane more readily [23] ultimately recycling carbon through this autotroph-heterotroph mutualism. The composition of carbon-degrading enzymes may therefore show whether chemolithoautotrophy or photosynthesis is more important for heterotrophic communities. Here, we focus on two broad classes of extracellular enzymes: peptidases which break down proteins and carbohydrate-active enzymes (CAZymes) which break down polysaccharides and related macromolecules.

**Fig 1:**
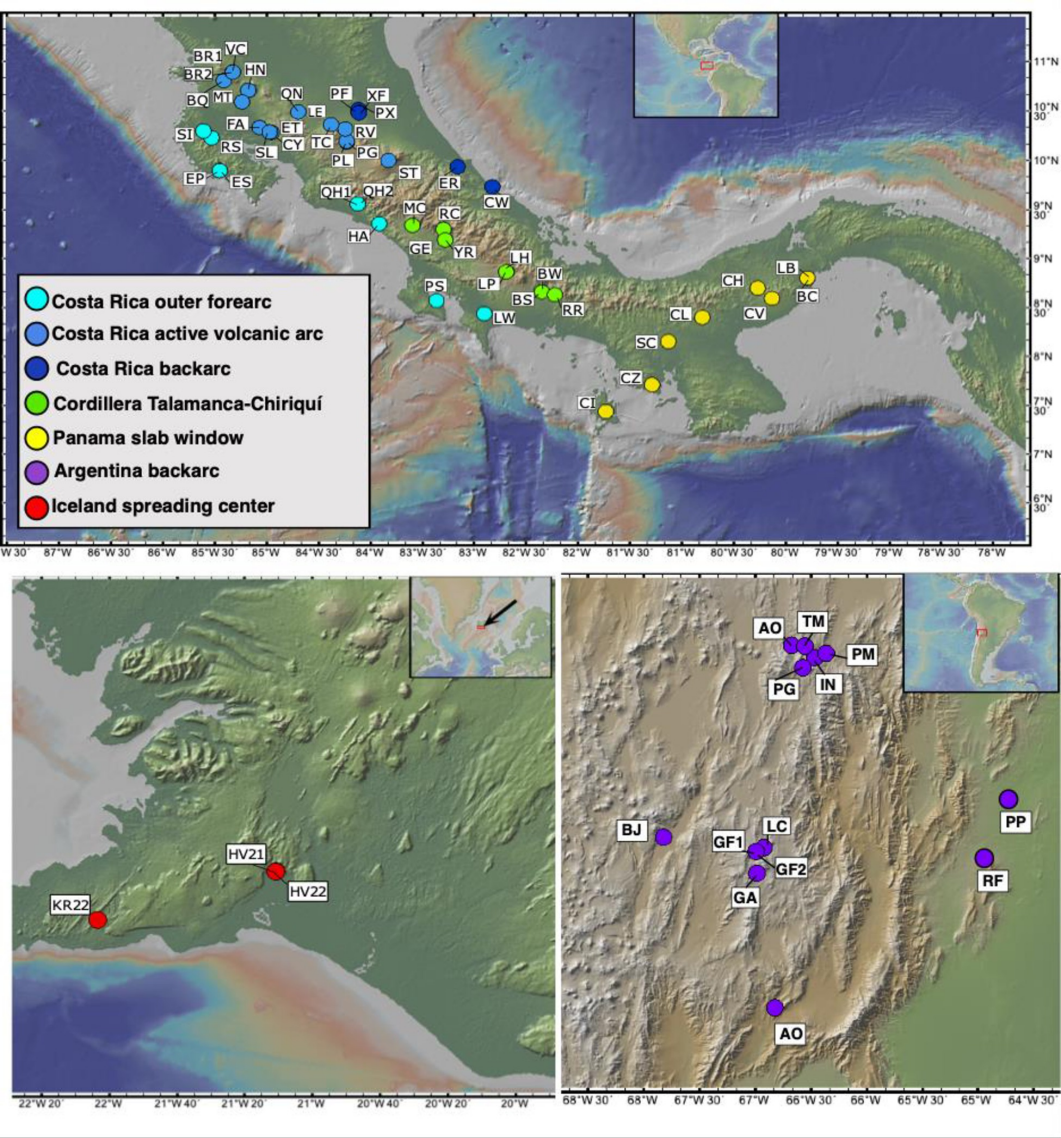
Maps of site locations. **A.** Costa Rica and Panama sites are color coded to match the different geological provinces. **B.** Iceland sites are all active volcanic hot spot and spreading center. **C.** Argentina sites are all backarc. Figure made with GeoMapApp (www.geomapapp.org) / CC BY / CC BY [31].

We compare springs across the Central American and the Andean (Argentinian backarc of the Central Volcanic Zone) convergent margins as well as the plume-influenced Iceland plate boundary. These regions are defined by their position across the convergent margin or continental intra-plate setting toward an oceanic plate boundary. The Costa Rican subduction zone is driven by the Cocos-Nazca plate subducting under the Caribbean plate. Northern Costa Rica is characterized by having higher volcanic activity than the other areas [24]. The Panama slab window is a result of a tear within the Nazca plate where arc volcanism ceases [25]. The formation of the slab window is also responsible for the cease in volcanism within the Cordillera Talamanca region [26], and a change in the rock chemistry in the area that shows hot spot-like compositions [27]. The Andean convergent margin is driven by the Nazca plate subducting under the South American plate [28], whereas Iceland is associated with spreading along the Mid-Atlantic Ridge under the influence of a mantle plume (e.g., [2, 29, 30]). Environmental factors also vary significantly between the sites due to large variations in latitude, elevation, and rainfall. This wide range of geological and environmental settings provides an opportunity to study geochemically diverse springs. Sites from Argentina and Iceland were placed into their own categories because they have different tectonic processes than those of Central America. The sites are then color coded based on these different geological processes that distinguish them (Fig 1).

Enzyme assays and bioinformatics analyses on metagenomes were used to analyze the microbial community interactions within diverse geothermal systems. To study the heterotrophic activity within geothermal systems, assemblies from the Costa Rican convergent margin, the Argentina backarc of the Andean convergent margin and the subaerial section of the Mid-Atlantic ridge (Iceland) were annotated using dbcan2 database and the run_dbcan package to find CAZymes and DRAM to annotate the MEROPS families [32, 33]. MEROPS classifies proteolytic enzymes using hierarchical classification by homologous sequences [34]. The CAZyme database splits carbohydrate-active enzymes into classes of glycoside hydrolases (GH), glycosyl transferases (GT), polysaccharide lyases (PL), carbohydrate esterases (CE), auxiliary activities (AA), and carbohydrate binding molecules (CBM), defined by sequence similarity [35]. Enzyme commission numbers are based on the reactions catalyzed instead of sequence homology [36]. Using these different tools allows for a broad analysis of the potential organic matter degrading functions of proteins based on their sequence homology. Hierarchical clustering and principal coordinate analysis were used to find correlations between the sites and the enzyme families found within them. By combining the maximum potential enzymatic activity, measured by low molecular mass fluorogenic substrate proxies, with the metagenomic annotations, we can see a larger scope of the potential heterotrophic activity within these sites.

## Materials and methods

### Sampling

DNA extraction and sequencing for all samples has been previously described [3, 4] Sample collection was performed following the protocols previously described [3, 4, 7] and using the rationale described by Giovannelli et al. 2022 [11]. Sites are color coded based on province (Fig 1) [31]. GPS coordinates, site names, temperature and pH measurements are shown in Table 1. Coauthors located at the University of Salta, Observatorio Volcanológico y Sismológico de Costa Rica (OVSICORI) Universidad Nacional, and NordVulk, Institute of Earth Sciences, University of Iceland, regularly visit these sites and required local verbal permission from landowners for each one of them. The samples we report here have been published previously [3, 4, 7, 37]. From each site, temperature and pH were measured directly in the fluids using a portable YSI Plus 6-Series Sonde Multimeter (YSI Incorporated, Yellow springs, OH) and 0.5 to 1.5 liters of hydrothermal fluids venting from the subsurface were collected. Care was taken when collecting fluids to do this as close to the perceived fluid source as possible. Fluids were immediately filtered through Sterivex 0.22 μm filter cartridges (MilliporeSigma) and quick-frozen onsite in a liquid nitrogen-cooled dry shipper. When fluid sampling was complete and to avoid resuspension, ~10 mL of surface sediments constantly overwashed by the venting source were placed into a sterile plastic vial and frozen onsite along with the filters. Sample names ending in (F) are from filtered fluids and those ending in (S) are from surface sediments.

**Table 1:** Site name and abbreviation supplemented with province, latitude, longitude, temperature, and pH. NM means not measured.

ABBREV.	SAMPLE NAME	SITE NAME	REGION	LAT	LONG	PROVINCE	TEMP °C	PH
**AO19**	AO190224	Antuco	Argentina	-24.182136	-66.674029	Argentina backarc	27.8	6.3
**AR17**	AR170220	Arenal Horse Farm	Costa Rica	10.4864	-84.6872	Costa Rica active volcanic arc	NM	NM
**BC18**	BC180410	Los Bajos the Corera	Panama	8.806037	-79.790968	Panama slab window	31.8	7.5
**BJ19**	BJ190227	Botijuela	Argentina	-25.743034	-67.823245	Argentina backarc	40	6.4
**BQ17**	BQ170218	Borinquen	Costa Rica	10.810883	-85.413707	Costa Rica active volcanic arc	88.9	2.1
**BR117**	BR170218_1	Blue River Spring 1	Costa Rica	10.89837	-85.32839	Costa Rica active volcanic arc	59	6.2
**BR217**	BR170218_2	Blue River Spring 2	Costa Rica	10.89837	-85.32853	Costa Rica active volcanic arc	53.8	5.9
**BS18**	BS180407	Bajo Mendez Spring	Costa Rica	8.66645	-82.3491	Cordillera Talamanca-Chiriquí	40.9	9.1
**BW18**	BW180407	Bajo Mendez Well	Costa Rica	8.66581	-82.34867	Cordillera Talamanca-Chiriquí	43.2	9.1
**CH18**	CH180410	Chiguiri Abajo	Panama	8.70508	-80.26919	Panama slab window	31.1	7
**CI18**	CI180408	Coiba Island	Panama	7.44104	-81.73277	Panama slab window	48.3	9
**CL18**	CL180409	Calobre	Panama	8.40448	-80.80375	Panama slab window	50.9	7.5
**CV18**	CV180410	Casa Valmor	Panama	8.5992	-80.13162	Panama slab window	34.9	7.5
**CW18**	CW180415	Cauhita Well	Costa Rica	9.735746	-82.825737	Costa Rica backarc	35	7.2
**CY17**	CY170214	Rio Cayuco	Costa Rica	10.287497	-84.955524	Costa Rica active volcanic arc	72	6.3
**CZ18**	CZ180409	Salitral Carrizal	Panama	7.71407	-81.28832	Panama slab window	26.3	10
**EP17**	EP170215	Espabel	Costa Rica	9.901885	-85.454327	Costa Rica outer forearc	26.4	9.9
**ES17**	ER180415	Rio Blanco Er Resbala	Costa Rica	9.938223	-83.161331	Costa Rica backarc	35	9.5
**ER18**	ES170215_1	Estrada	Costa Rica	9.899005	-85.453514	Costa Rica outer forearc	27.9	9.7
**ET17**	ET170220_1	Eco Thermales	Costa Rica	10.484006	-84.675853	Costa Rica active volcanic arc	40	6.1
**FA17**	FA170219_1	Finca Ande	Costa Rica	10.336843	-85.069499	Costa Rica active volcanic arc	55.2	5.9
**GA19**	GA190226	Galán Aguas Calientes	Argentina	-25.825416	-66.922496	Argentina backarc	67	6.7
**GE18**	GE180403	Gevi	Costa Rica	9.19483333	-83.280806	Cordillera Talamanca-Chiriquí	35.8	7.8
**GF119**	GF190226_1	Galán Fumaroles 1	Argentina	-25.858188	-66.992695	Argentina backarc	80	7.8
**GF219**	GF190226_2	Galán Fumaroles 2	Argentina	-25.858243	-66.992818	Argentina backarc	80	3.2
**HA18**	HA180403	Hattillo	Costa Rica	9.36022	-83.91664	Cordillera Talamanca-Chiriquí	33	8.9
**HV121**	HV1210602	Hveragerdi 1	Iceland	64.008117	-21.17949	Iceland spreading center	93.5	2.7
**HV221**	HV2210602	Hveragerdi 2	Iceland	64.007062	-21.180739	Iceland spreading center	25.7	1.8
**IN19**	IN190223	Incachule	Argentina	-24.282129	-66.466761	Argentina backarc	46.9	6.5
**KR21**	KR2210530	Krysuvik upper pool	Iceland	63.895451	-22.057004	Iceland spreading center	93	2
**LB18**	LB180410	Los Bajos	Panama	8.80736	-79.79061	Panama slab window	34.8	NM
**LC19**	LC190226	Galán La Colcha	Argentina	-26.032911	-66.986094	Argentina backarc	84	6.9
**LE18**	LE180416	Las Estrella	Costa Rica	10.427103	-84.368543	Cordillera Talamanca-Chiriquí	34.7	NM
**LH18**	LH180406	Los Pozos Thermales	Panama	8.87095	-82.6899	Cordillera Talamanca-Chiriquí	55.4	6.7
**LP18**	LP180406	Los Pozos Thermales	Panama	8.86966	-82.69282	Cordillera Talamanca-Chiriquí	39.1	6.5
**LW18**	LW180405	Laurel	Costa Rica	8.44119	-82.90487	Costa Rica outer forearc	31.5	7.1
**MC18**	MC180404	Montecarlo—Bernardino	Costa Rica	9.34391	-83.59565	Cordillera Talamanca-Chiriquí	31.8	9.6
**MT17**	MT170219	Termales Salitral	Costa Rica	10.595774	-85.238451	Costa Rica active volcanic arc	59.1	6.3
**PB17**	PB170224	Poas Volcano background soil	Costa Rica	10.196777	-84.229892	Costa Rica active volcanic arc	NM	NM
**PF17**	PF170222	Pompilo’s finca	Costa Rica	10.518466	-84.11518	Costa Rica backarc	28.7	5.8
**PG19**	PG190225	Pastos Grandes	Argentina	-24.364589	-66.571132	Argentina backarc	44.9	8.7
**PG17**	PG172224	Poas Volcano Laguna	Costa Rica	10.188962	-84.227388	Costa Rica active volcanic arc	19.2	NM
**PL17**	PL170224	Poas Volcano Lake	Costa Rica	10.196777	-84.229892	Costa Rica active volcanic arc	37.6	0.8
**PM19**	PM190223	Pompeya	Argentina	-24.246688	-66.362722	Argentina backarc	50.3	6.5
**PP19**	PP190301	El Galpón Pio Perez	Argentina	-24.40986	-64.59146	Argentina backarc	54.3	8.5
**PS18**	PS180405	Playa Sandalo	Costa Rica	8.57554	-83.36416	Costa Rica outer forearc	33	8.2
**PX18**	PX180416	Praxair well 24	Costa Rica	10.488755	-84.113598	Costa Rica backarc	28.7	NM
**QH117**	QH170213_1	Quepos Hot springs	Costa Rica	9.56171	-84.123251	Costa Rica outer forearc	48.7	8.5
**QH217**	QH170213_2	Quepos Hot springs	Costa Rica	9.561575	-84.123468	Costa Rica outer forearc	36.7	8.7
**QN17**	QN170220	Quebrada naranja	Costa Rica	10.495573	-84.696714	Costa Rica active volcanic arc	22.9	5.6
**RC18**	RC180404	Ujarassa	Costa Rica	9.30283	-83.29782	Cordillera Talamanca-Chiriquí	60	7.7
**RF19**	RF190301	Rosario de la Frontera	Argentina	-25.40986	-64.59134	Argentina backarc	82	8.2
**RR18**	RR180407	"Rockslide"	Costa Rica	8.63591	-82.22369	Cordillera Talamanca-Chiriquí	41.3	NM
**RS17**	RS170216	Ranchero etl Salitral	Costa Rica	10.232331	-85.531602	Costa Rica outer forearc	29.4	9.9
**RV17**	RV170221	Recreo Verde	Costa Rica	10.321576	-84.243686	Costa Rica active volcanic arc	42.7	6.2
**SC18**	SC180411	El Salao Campollano	Panama	8.15755	-81.13097	Panama slab window	29.9	7
**SI17**	SI170217	El Sitio	Costa Rica	10.301239	-85.610549	Costa Rica outer forearc	35.9	9.8
**SL17**	SL170214	Santa Lucia	Costa Rica	10.290599	-84.972435	Costa Rica active volcanic arc	57	6.1
**TC17**	TC170221	El Tucano bubbling site	Costa Rica	10.366486	-84.381208	Costa Rica active volcanic arc	60	6.2
**TM19**	TM190224	Tocomar	Argentina	-24.18778	-66.55451	Argentina backarc	69.2	7.1
**VV19**	VV190228	Villa Vil	Argentina	-27.112858	-66.822241	Argentina backarc	38.2	9.1
**XF18**	XF180416	Praxair well 19	Costa Rica	10.485523	-84.113229	Costa Rica backarc	28.9	7
**YR18**	YR180404	Yheri	Costa Rica	9.19492	-83.28059	Cordillera Talamanca-Chiriquí	26	8.9

### Bioinformatic processing

For the Iceland metagenomes, raw reads were trimmed with Trimmomatic (v 0.39) [38] and assembled using the MetaWRAP (v 1.3.2) pipeline [39]. The quality of the Iceland assemblies was determined using Quast (v4.4) [40] on Kbase [41]). All other assemblies were generated by trimming raw reads with Trimmomatic (v 0.38). Reads were assembled *de novo* with metaSPAdes with a minimum contig length of 1.5 kb [7]. Reads from LC19F, LC19S, RF19S, and TM19S were assembled using MEGAHIT (v 1.2.9) [42]. The assemblies were annotated using prokka (v 1.14.5) [43] and dbcan2 [32]. For peptidase annotations the assemblies were uploaded to KBase [41] and annotated with DRAM (v.0.1.0) [33]. Secreted proteins were identified using SignalP (v 2.0) [44]. The clean reads were mapped back to the assemblies for read coverage using bowtie2 (v 2.3.5.1) and samtools (v1.15.1) [45, 46]. Hierarchical clustering based on spearman correlation, was performed using hclust from the base R package (R version 4.2.1). Total microbial community analysis of these sites is the focus of previous work, therefore, taxonomic identification was only performed for contigs for the high temperature sites HV121S, HV221S, and KR21S using gottcha2 on Kbase (S1 Fig in S1 File) [4, 7, 47–49].

CAZy family abundances were annotated using dbcan2. Annotations were selected if they were annotated with at least two tools: HMMER and DIAMOND. The annotated gene IDs were then combined with the prokka gene ID and contigs to gain the abundance of each contig. Read coverage was calculated as previously described [7] Then the read coverage of each annotation normalized to the total assembly size was used to generate a heatmap with hierarchical clustering of the CAZyme families and site locations. All annotations that are presented also were annotated for having a signal peptide sequence by SignalP [44]. Protein annotations that are not present within 75% of the assemblies were removed for better visualization.

Enzyme commission numbers were assigned with dbcan2 database and combined with the SignalP annotations to estimate secretion. Enzyme commission groupings are only shown for class 3 hydrolases where the enzyme commission number was present in at least 75% of the assemblies. MEROPS peptidase annotations were completed using DRAM on Kbase and annotated using SignalP. The gene IDs were then matched to contigs of the assemblies to get the normalized read coverage.

### Enzymatic assays

Enzymatic assays were performed following the methods of Bell (2013) [50] with slight modifications. Briefly, sediments were weighed out at 2.75 grams wet weight. The sediments were then combined with 91 mL of 0.5 M Tris-HCl buffer with a matching pH to the original site locations. The sediment slurries were blended for one minute to homogenize them. Then 800 μL of each slurry was pipetted into deep well plates in duplicate. 200 μL of substrates with a concentration of 200 μM were then added to each well. The substrates used were 4-Methylumbelliferyl α-D-glucopyranoside (AG), 4-Methylumbelliferyl β-D-glucopyranoside (BG), 4-Methylumbelliferyl β-D-cellobiosidase (CB), 4-Methylumbelliferyl N-acetyl-β-d-glucosaminidase (NAG), L-Leucine-7-amido-4-methylcoumarin (LEU), 4-Methylumbelliferyl phosphate (PHOS), 4-Methylumbelliferyl sulfate potassium salt (SULF), 4-Methylumbelliferyl-β-D-xylopyranoside (XYL). Each deep well plate was incubated for 3 hours at 30–70°C, and time points were taken at 0 hours, 1.5 hours, and 3 hours. To take each time point, 200 μL was pipetted from the deep well plate to a black flat bottom 96 well plate. The fluorescence was measured at 455 nm after excitation at 355 nm using a Tecan Infinite M200 Pro Fluorimeter. Differences in enzyme activities among provinces were tested using a Kruskal-Wallis test, implemented in R, due to the strong non-normal distribution of activities in the data set.

## Results

### Sites

Three different geographical areas were analyzed: the Central American and Andean convergent margins, and Iceland (mantle plume-influenced spreading center). Large variations in fluid sources (i.e. mantle, slab, crust, or surficial) are expected due to the contrasting geologic and environmental settings of the studied areas. We include data from 22 sites in Costa Rica that are influenced by the convergent margin, with analyses of the geochemistry, respirations, and taxonomic identities of the 22 sites published previously [3, 4, 7, 51]. We include 47 additional sites spanning Costa Rica and Panama that are also influenced by the convergent margin, 13 sites from the Andean convergent margin and 3 sites from Iceland. Sites were grouped by geographic-tectonic setting: Costa Rica outer forearc, Costa Rica active volcanic arc, Costa Rica backarc, Cordillera Talamanca, Panama, Argentina backarc, and Iceland (Fig 1, Table 1). These groupings allow us to explore large variations in fluid sources and physicochemical characteristics which are ultimately related to their tectonic setting. For example, in Costa Rica the distance to the trench (outer forearc to active arc to backarc) correlates with large variations in temperature, pH, and mantle-derived components that affect chemical compositions of the fluids and the microbial communities [3, 4]. In total 13 sites have the lowest temperatures (19.2–29.9°C) and eight sites have the highest temperatures (80–93.5°C) (Table 1). Seven sites have the lowest pH (0.85–3.21), while 11 sites have the highest pH (9.0–10.0).

### Enzyme activities

**Fig 2:**
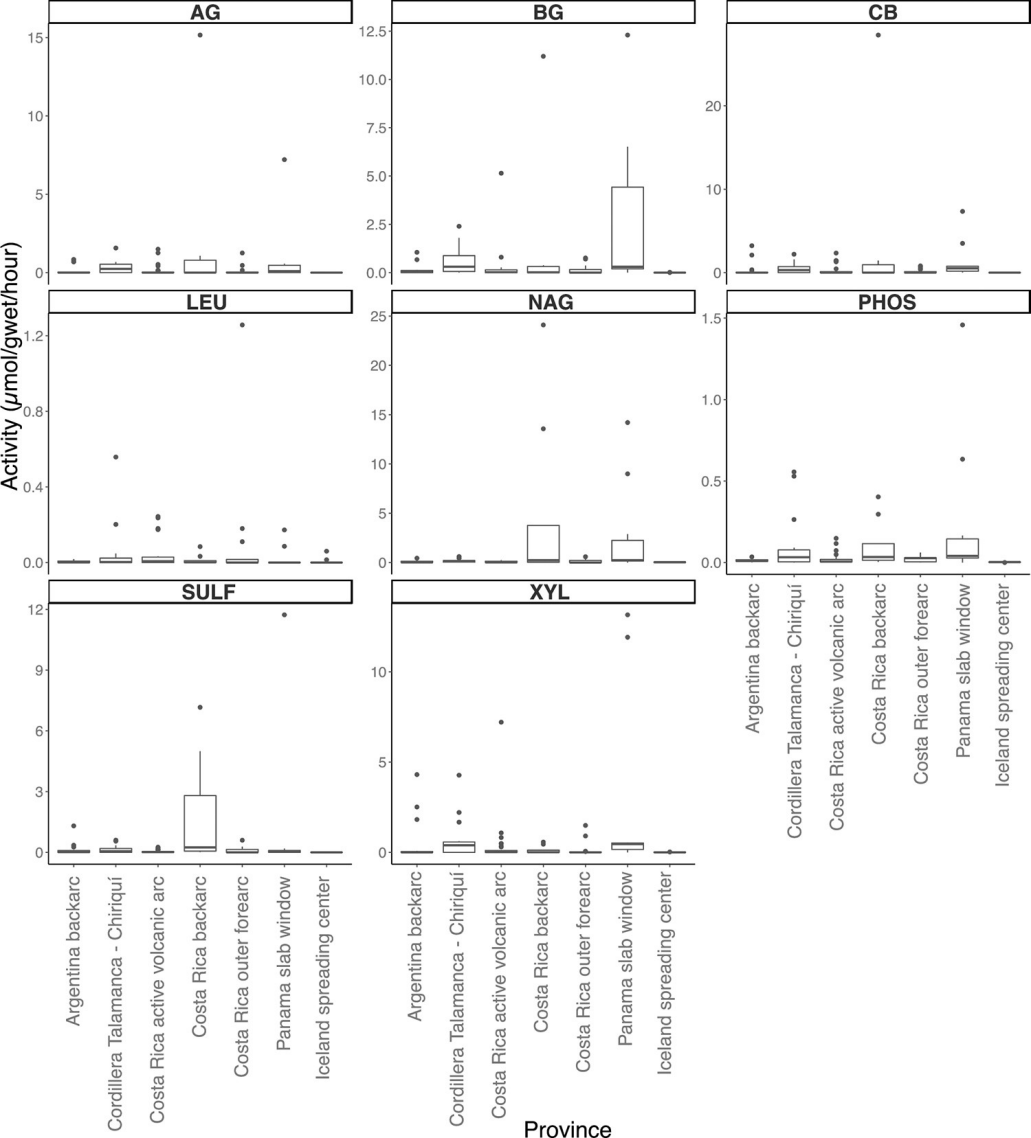
Enzyme activity box plot. Activities for each site were grouped together based on province. Activity measurements were only performed on sediment samples. Each activity is presented in μmol/g_wet_/h. Enzyme substrates were separated into different panels. Enzymes abbreviations are alpha-glucosidase (AG), beta-glucosidase (BG), cellobiohydrolase (CB), leucine aminopeptidase (LEU), N-acetyl-β-D-glucosaminidase (NAG), phosphatase (PHOS), sulfatase (SULF), and xylosidase (XYL).

Most of the hydrolysis rates measured were indistinguishable from zero (Fig 2). This could mean either that few of the enzymes were being expressed at the time of sampling or that we were unable to properly recreate the geochemical conditions present *in situ*. Of the enzymes tested, carbon-acquiring enzymes (AG, BG, CB, XYL) were more active than enzymes associated with phosphorus and sulfur acquisition (PHOS and SULF) (S3 Table in S1 File). LEU hydrolysis was orders of magnitude lower than the other enzymes assayed, in contrast to soils where LEU often has high hydrolysis rates [52]. NAG hydrolysis was positive at more sites than the other substrates. PHOS hydrolysis had only one site with zero activity, CL18S. From the Kruskal-Wallis statistical analysis, no significant differences (p>0.05) were observed for any enzyme activities between provinces.

### Metagenomic assemblies

Of the three metagenomic assemblies from Iceland, HV221S has 4,344 contigs, KR21S has 3,214, and HV121S has 912 (S1 Table in S1 File). The total length of these assemblies is 5,645,364 bp for HV121S, 7,802,221 bp for HV221S, and 12,561,178 bp for KR22. The total contig numbers for the Costa Rica, Panama and Argentina assemblies range from 488 to 164,798 (S2 Table in S1 File). The total sizes of the Costa Rica, Panama and Argentina assemblies range from 8,678,655 to 509,373,734 (S2 Table in S1 File). Genus level taxonomy classification was done using gottcha2 on Kbase (S1 Fig in S1 File) [41, 47]. At the genus level, HV121S has 70% of reads annotated as *Sulfolobus*, 15% as *Acidianus*, and the 8% as *Thermoproteus*.The remaining reads are distributed across *Methylorubrum*, *Pseudomonas*, and *Stenotrophomonas*. HV21S had 38% of reads annotated as *Sulfolobus*, 21% as *Thermoproteus*, 10% as *Cutibacterium*, and the rest are distributed across other genera. KR21S has 45% of reads identified as *Acidianus*, 8% as *Sulfolobus*, and 5% as *Thermoproteus*, with the remaining taxonomy distributed across other genera.

### Peptidases

**Fig 3:**
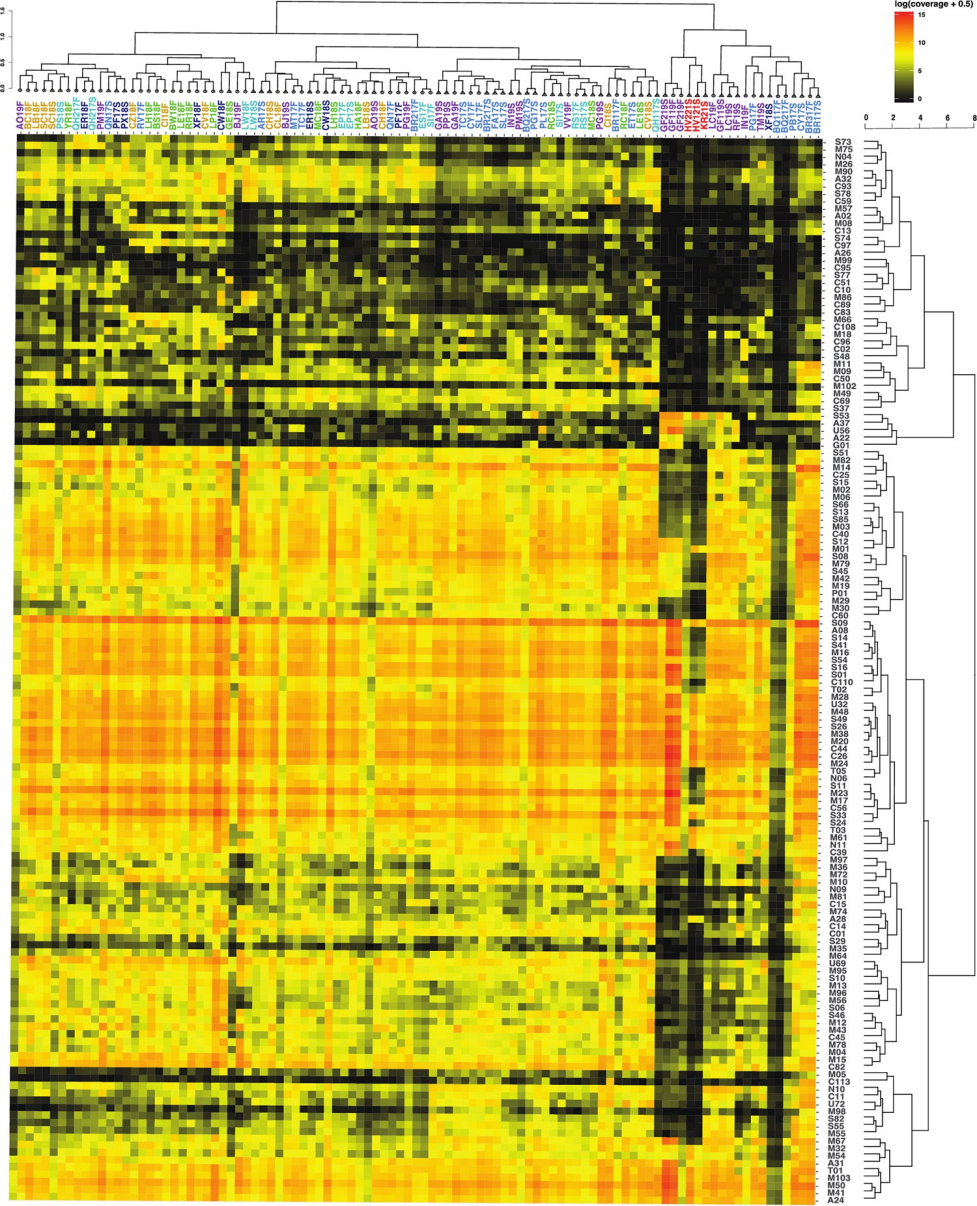
Heatmap with hierarchical correlation of read abundances of MEROPS families (y axis) per site (x axis) based on spearman rank correlation. Abundance shown in log of normalized contig abundance with 0.5 added to avoid zeros for visualization. Untransformed abundances were used for the spearman correlation. Sites are colored by the geological provinces shown in Fig 1.

In total, there are 144 MEROPS family annotations predicted to be secreted (Fig 3), M (Metallo) with 59, S (Serine) with 32, C (Cysteine) with 29, A (Aspartic) with 9, N (Asparagine) with 5, T (Threonine) with 4, U (Unknown) with 4, G (Glutamic) with 1, and P (Mixed) with 1 (Fig 3). Of these 144 families, 88 are present in every assembly. The total read abundance of annotations for MEROPS is 283,488,080, which is much greater than those of CAZy at 1,729,668, and EC3 at 633,511, which are discussed below. Important MEROPS families are listed in Table 2.

**Table 2:** CAZy and MEROPS families listed based on the substrate group preference. The groups associated with cell degradation are chitin and peptidoglycan. Photosynthate degradation groups are xylan and cellulose [53–59].

GROUP	CAZY FAMILY	MEROPS FAMILY
**CHITIN**	GH5, GH7, GH8, GH18, GH19, GH20, GH46, CBM5	
**PEPTIDOGLYCAN**	GH22, GH23, GH24, GH25, GH102, GH103, GH108, CBM50, CE4	S11, M23, S13, S66, M15, M74, M14, C51
**STARCH/** **GLYCOGEN**	GH3, GH13, GH14, GH15, GH27, GH31, GH38, GH57, GH72, GH77, GH89, GH119, GH126, AA13, GT35, CBM20	
**TREHALOSE**	GH37, GH65	
**XYLAN**	GH3, GH5, GH7, GH8, GH10, GH11, GH43, GH67	
**CELLULOSE**	GH1, GH3, GH5, GH8, GH9, GH10, GH16, GH43, GH51, GH74, GH116	

The highest read abundance normalized to total assembly size is 5,316,108.99 for M23 in site GF1f. The most abundant MEROPS normalized read abundances are S09 (prolyl oligopeptidase), M23 (beta-lytic metallopeptidase), C26 (gamma-glutamyl hydrolase), S33 (prolyl aminopeptidase), M38 (isoaspartyl dipeptidase), C44 (amidophosphoribosyl transferase precursor), S49 (signal peptide peptidase A), M20 (glutamate carboxypeptidase), M50 (site 2 peptidase), and S16 (Lon-A peptidase). The sites with the least amount of MEROPS annotations are GF219F, HV121S, GF219S, and GF119F.

Within the spearman correlation hierarchical clustering of sites based on the MEROPS families, the highest temperature sites (GF219S, GF119F, GF219F, HV221S, HV121S, and KR21S) cluster together. Some sites’ sediment and fluid assemblies cluster together: SC18F, LW18F, EP17F, RS17F, and BR117F. One cluster consists of only fluid samples CZ18F, RV17F, LH18F, BS18F, CI18F, BW18F, LE18F, RR18F, XF18F, CV18F, and LP18F. For the hierarchical clustering of MEROPS families based on their distribution across sites, one cluster contains MEROPS families S53, A37, U56, A22, G01, that are highly present in only the very hot sites. It has been shown that these peptidase families are associated with acidophilic or thermophilic archaea [60].

### CAZymes

CAZy families predicted to be secreted are present in all sites at orders of magnitude lower read abundance than peptidases. Of the six CAZy classes, the most abundant are the glycosyl hydrolases (GH). CAZy families that are abundant in all sites are GH23 (peptidoglycan lyase), CBM50 (binds peptidoglycan and chitin), GH102 (peptidoglycan lytic transglycosylase), and GH103 (peptidoglycan lytic transglycosylase). All five CAZy classes are present in 76 of the assemblies with 89 annotations from glycoside hydrolases (GH), 31 from carbohydrate binding modules (CBM), 17 from polysaccharide lyases (PL), 10 from carbohydrate esterases (CE), 7 from glycosyltransferases (GT), and 2 from auxiliary activities (AA). There are a total of 156 CAZy families present. The assemblies with the least number of CAZyme annotations are HV121S, KR21S, GF119F, GF219F, GF219S and BQ217F. Sites BQ117F, BQ217F, GF119F, GF219F, GF219S, HV121S, KR21S, LC19s, and RF19S, had less than 75% of the total CAZyme annotations presented, all of which had temperatures over 80°C.

**Fig 4:**
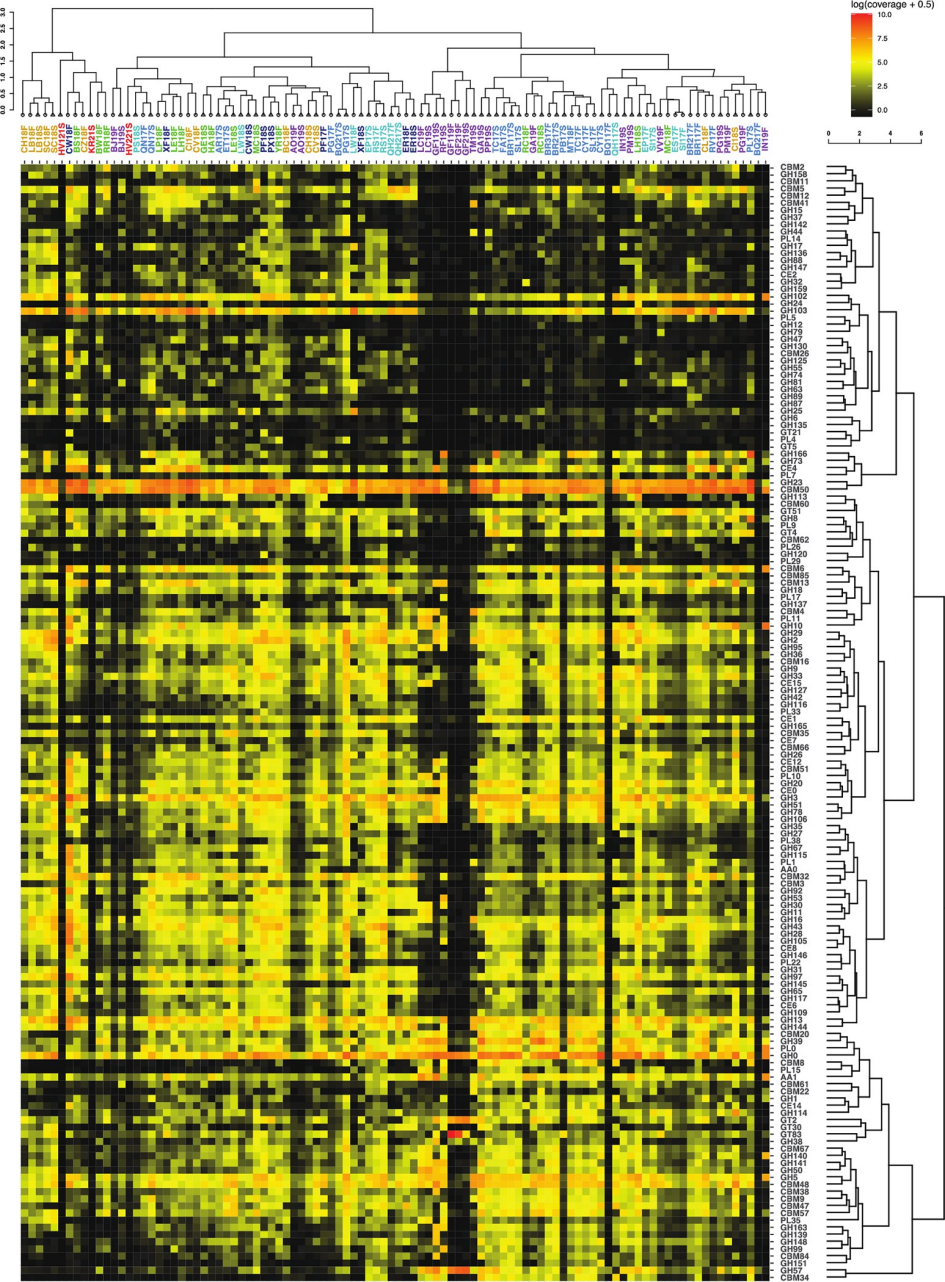
Heatmap with hierarchical correlation of read abundance of CAZy families (y axis) per site (x axis) based on spearman rank correlation. Visualization details are the same as Fig 3.

Within the spearman correlation of the site clustering, we see a grouping of sites that are from the Argentina backarc and have a higher temperature range (Fig 4). These sites cluster together due to the low abundance of enzyme annotations within their assemblies. Some sites’ sediment and fluid samples cluster together such as, LB18, SC18, BJ19, QN17, AO19, RS17, QH217, ER18, and LC19.

### Enzyme commission

**Fig 5:**
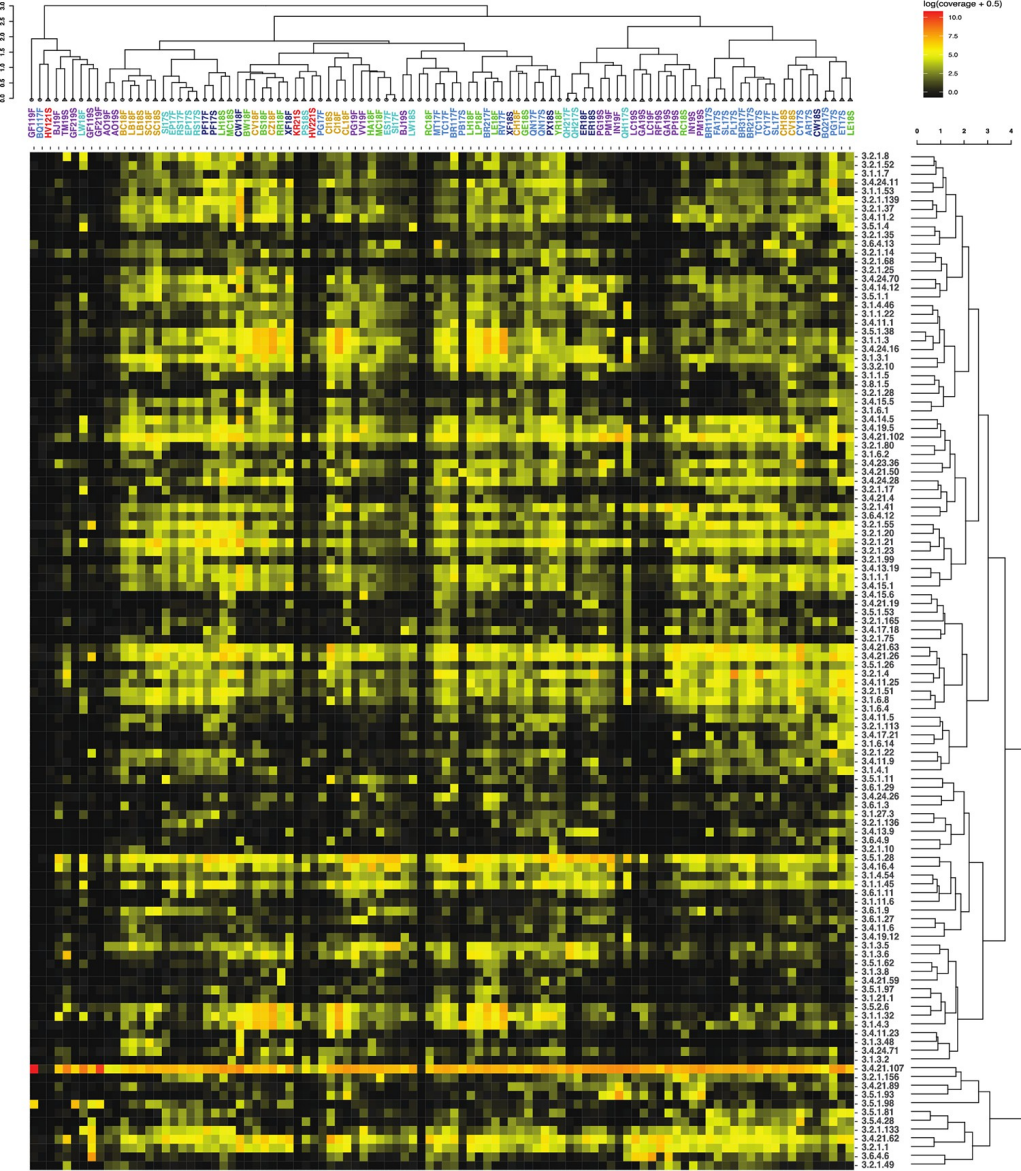
Heatmap with hierarchical correlation of read abundance of hydrolases (EC 3, y axis) per site, based on spearman rank correlation. Visualization details are the same as Fig 3.

Enzymes from EC3 are hydrolases, so many of them overlap with those in the CAZyme and MEROPS groups, but the EC categories are more finely divided than CAZyme groups, so they describe hydrolase functionality more precisely. As with CAZymes, EC3 annotations (0–49,251.04) have orders of magnitude lower read abundance than the MEROPs peptidases (0–5,316,108.99) (Fig 5). There are a total of 117 EC hydrolase annotations. The EC hydrolases are subdivided into 3.1 (ester hydrolases), 3.2 (glycosylases), 3.3 (ether hydrolases), 3.4 (peptidases), 3.5 (other non-peptide carbon and nitrogen hydrolases), 3.6 (acid anhydride hydrolases), 3.7 (other carbon-carbon bond hydrolases), 3.8 (halide hydrolases), 3.9 (other phosphorus nitrogen bond hydrolases), 3.10 (sulfur nitrogen bond hydrolases), 3.11 (other carbon phosphorus bond hydrolases), 3.12 (sulfur-sulfur bond hydrolases), 3.13 (carbon sulfur bond hydrolases). The spearman site correlation shows a clustering of sites with very few hydrolases present (Fig 5). The sites that have less than 75% of the protein annotations shown are IN19F, LC19F, HV221S, RF19S, BQ117F, GF119S, BQ217F, KR21S, HV121S, LC19S, GF219S, GF219F, and GF119F. All these sites except for IN19 fall within the highest temperature range (80–93.5°C). The most abundant EC annotation is 3.4.21.107 (peptidase Do). Other ECs in high abundance are N-acetylmuramoyl-L-alanine amidase (3.5.1.28), subtilisin (3.4.21.62), C-terminal processing peptidase (3.4.21.102), prolyl oligopeptidase (3.4.21.26), oryzin (3.4.21.63), triacylglycerol lipase (3.1.1.3), and beta-lactamase (3.5.2.6). The most common category among these high abundance enzymes is EC group 3.4, which are peptidases.

The EC numbers that are present in all assemblies are alkaline phosphatase (3.1.3.1), (3.2.1.1), beta-glucosidase (3.2.1.21), alpha-L-fucosidase (3.2.1.51), dipeptidyl-peptidase IV (3.4.14.5), peptidyl-dipeptidase A (3.4.15.1), peptidyl-dipeptidase Dcp (3.4.15.5), peptidase Do (3.4.21.107), subtilisin (3.4.21.62), oryzin (3.4.21.63), and endothelin-converting enzyme 1 (3.4.24.71). Three of the EC numbers that are present in all assemblies are also some of the most abundant (3.4.21.107, 3.4.21.62, and 3.4.21.63). Of the eleven EC numbers present in all assemblies, seven are within the family of peptidases. As with the MEROPS and CAZymes annotations, some of the fluid and sediments from the same site group together: AO19, LB18, SC18, PF17, CI18, QN17, QH217, and ER18.

### CAZy degradation groupings

**Fig 6:**
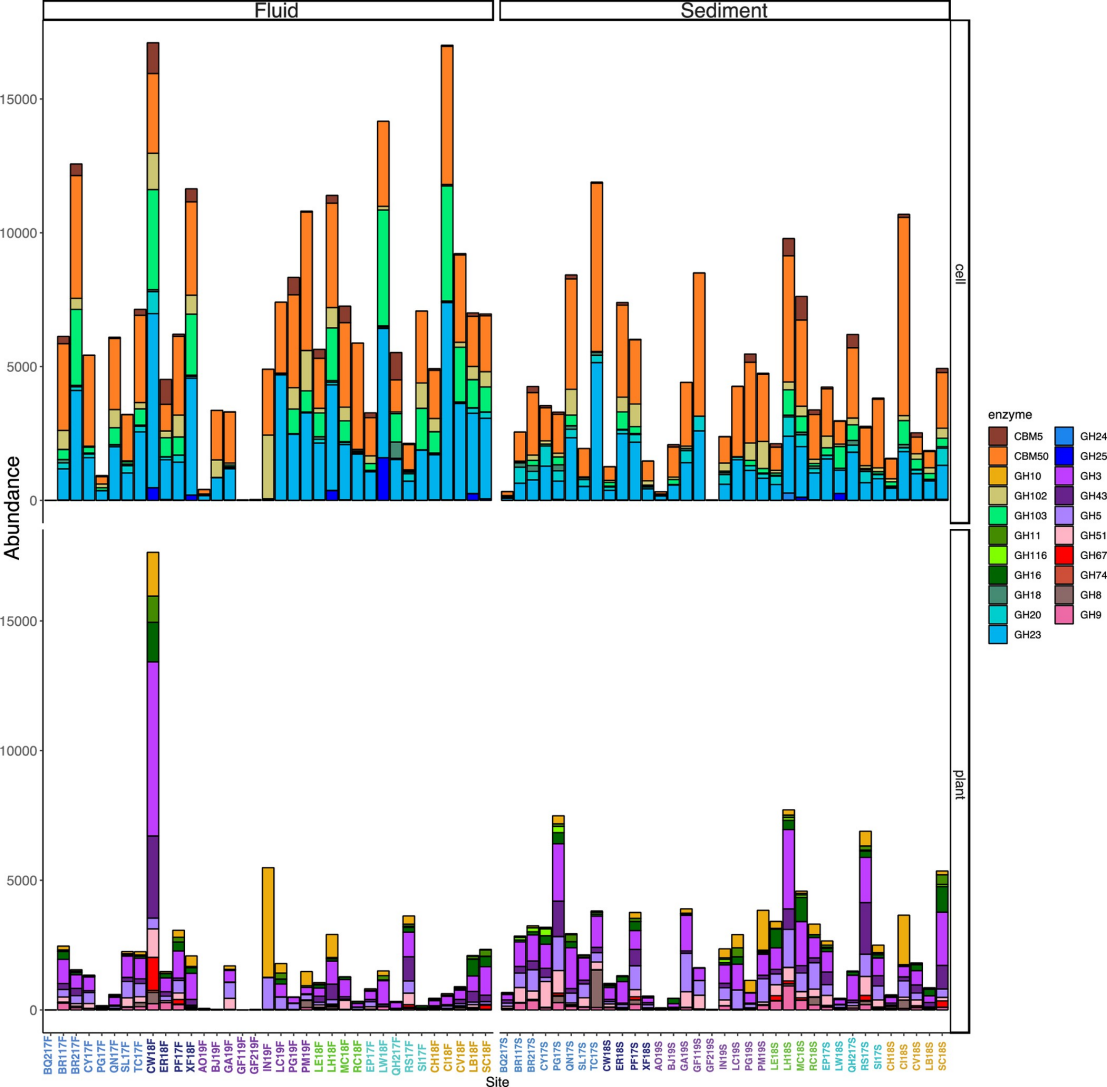
Read abundance of cell-degrading and photosynthate-degrading enzymes in metagenomic assemblies from sediments and fluids, with sites separated by geological province. Sites are colored by the geological provinces shown in Fig 1.

We summed the abundance of CAZy families that are either part of chitin/peptidoglycan degradation (bacterial necromass) or xylan degradation (plant products) (Tables 2 and 3). In total cell-degrading enzymes are in higher read abundance (521,794.37) than photosynthate-degrading enzymes (227,434.98) (Table 3). The assemblies with the highest percentage of cell-degrading enzymes are BJ18F, SI17F, KR21S, ES17F, RV17F, and CI18F. Only 20 assemblies have fewer than 50% of the cell-degrading enzyme annotations. The abundance of these enzymes for each site, with sediment and fluid components, is shown in a stacked bar plot to visualize the differing quantity across sediments and fluids (Fig 6). Overall, cell-degrading enzymes are more abundant than photosynthate-degrading enzymes. Photosynthate-degrading enzymes are more abundant in sediments than in fluids, whereas the cell-degrading enzymes are more abundant in fluids than in sediments (Table 3). CAZy family GH103 is more abundant in fluids than in sediments (Fig 6). CAZy families GH18 and GH3 are more abundant in sediments than fluids.

**Table 3:** Sums of enzymes associated with cell and photosynthate degradation. Cell enzymes are in the chitin and peptidoglycan groups of Table 3. The photosynthate enzymes are in the groups xylan and cellulose of Table 3. The total of these two groups are summed for each site.

Site	Cell	Plant	Province	Type	Temp °C	pH	% cell degradation
**RS17S**	2303.35	7311.38	Costa Rica outer forearc	Sediment	29.4	9.96	0.24
**PG17S**	2717.43	7824.97	Active volcanic arc	Sediment	19.2		0.26
**BQ217S**	275.26	702.8	Active volcanic arc	Sediment	88.9	2.11	0.28
**FA17S**	1356.01	3157.92	Active volcanic arc	Sediment	55.2	5.93	0.3
**RS17F**	1793.86	3927.14	Costa Rica outer forearc	Fluid	29.4	9.96	0.31
**LE18S**	1779.26	3744.37	Cordillera Talamanca	Sediment	34.7		0.32
**BR117S**	1790.83	3507.56	Active volcanic arc	Sediment	53.8	5.87	0.34
**SL17S**	1608.47	2395.79	Active volcanic arc	Sediment	57	6.12	0.4
**SC18S**	4231.4	5974.49	Panama slab window	Sediment	29.9	6.5	0.41
**IN19S**	1951.11	2651.99	Argentina backarc	Sediment	46.9	6.52	0.42
**CY17S**	2782.04	3678.12	Active volcanic arc	Sediment	72	6.31	0.43
**BR317F**	2259.54	2880.35	Active volcanic arc	Fluid	59	6.16	0.44
**PP19S**	2768.98	3466.71	Argentina backarc	Sediment	54.3	8.47	0.44
**YR18F**	3699.46	4556.12	Cordillera Talamanca	Fluid	26	8.9	0.45
**RC18S**	3124.49	3563.44	Cordillera Talamanca	Sediment	60	7.7	0.47
**GA19S**	3880.17	4421.99	Argentina backarc	Sediment	67	6.7	0.47
**CW18F**	16280.11	18463.25	Active volcanic backarc	Fluid	35	7.19	0.47
**IN19F**	4897.84	5482.85	Argentina backarc	Fluid	46.9	6.52	0.47
**GF219S**	8.21	8.62	Argentina backarc	Sediment	80	3.21	0.49
**CW18S**	1102.96	1140.82	Active volcanic backarc	Sediment	35	7.19	0.49
**ET17S**	3686.85	3635.11	Active volcanic arc	Sediment	40	6.06	0.5
**BR217S**	3714.33	3629.83	Active volcanic arc	Sediment	53.8	5.87	0.51
**LH18S**	8995.25	8393.51	Cordillera Talamanca	Sediment	55.4	6.7	0.52
**SL17F**	2882.97	2547.03	Active volcanic arc	Fluid	57	6.12	0.53
**PM19S**	4557.13	3980.86	Argentina backarc	Sediment	50.3	6.53	0.53
**CV18S**	2364.68	1971.3	Panama slab window	Sediment	34.9	7.46	0.55
**RF19S**	2319.97	1777.15	Argentina backarc	Sediment	82	8.23	0.57
**GE18S**	2650.99	1939.12	Cordillera Talamanca	Sediment	35.8	7.8	0.58
**LC19S**	4152.17	3007.55	Argentina backarc	Sediment	84	6.94	0.58
**PF17S**	5696.03	4077.12	Active volcanic backarc	Sediment	28.7	5.81	0.58
**SI17S**	3655.49	2607.84	Costa Rica outer forearc	Sediment	35.9	9.83	0.58
**MC18S**	7088.09	5020.36	Cordillera Talamanca	Sediment	31.8	9.6	0.59
**HV121S**	5.04	3.52	Spreading center hot spot	Sediment	93.5	2.72	0.59
**EP17S**	4095.65	2760.82	Costa Rica outer forearc	Sediment	26.4	9.99	0.6
**AR17S**	1015.94	613.94	Active volcanic arc	Sediment			0.62
**PF17F**	5945.38	3326.85	Active volcanic backarc	Fluid	28.7	5.81	0.64
**GA19F**	3232.45	1772.08	Argentina backarc	Fluid	67	6.7	0.65
**LB18S**	1799.92	907.5	Panama slab window	Sediment	34.8	5.951	0.66
**RR18F**	2427.99	1216.93	Cordillera Talamanca	Fluid	41.3	7.2756	0.67
**BR117F**	5777.06	2784.88	Active volcanic arc	Fluid	59	6.16	0.67
**PX18S**	7241.69	3328.99	Active volcanic backarc	Sediment	42.9225	6.3077	0.69
**XF18S**	1378.69	617.31	Active volcanic backarc	Sediment	28.9	7	0.69
**CH18S**	1497.87	644.82	Panama slab window	Sediment	31.1	7	0.7
**QN17S**	8016.9	3343.38	Active volcanic arc	Sediment	22.9	5.6	0.71
**VV19F**	4672.04	1821.79	Argentina backarc	Fluid	38.2	9.09	0.72
**SC18F**	6730.07	2555.04	Panama slab window	Fluid	29.9	6.5	0.72
**BC18F**	3840.09	1452.1	Panama slab window	Fluid	31.8	7.5	0.73
**CI18S**	10418.56	3918.02	Panama slab window	Sediment	48.3	9	0.73
**BS18F**	9438.61	3449.51	Cordillera Talamanca	Fluid	40.9	9.05	0.73
**TC17S**	11507.98	4132.12	Active volcanic arc	Sediment	60	6.24	0.74
**ER18F**	4408.88	1580.28	Active volcanic backarc	Fluid	35	9.51	0.74
**TC17F**	6889.35	2454.24	Active volcanic arc	Fluid	60	6.24	0.74
**BQ117F**	4.55	1.61	Active volcanic arc	Fluid	88.9	2.11	0.74
**LB18F**	6797.71	2293.94	Panama slab window	Fluid	34.8	5.951	0.75
**QH217S**	5764.17	1913.66	Costa Rica outer forearc	Sediment	36.7	8.69	0.75
**CL18F**	10123.52	3243.36	Panama slab window	Fluid	50.9	7.5	0.76
**MT17F**	7584.31	2352.33	Active volcanic arc	Fluid	59.1	6.32	0.76
**CY17F**	5257.73	1501	Active volcanic arc	Fluid	72	6.31	0.78
**TM19S**	10956.68	3105.54	Argentina backarc	Sediment	69.2	7.13	0.78
**QH117S**	5565.26	1527.88	Costa Rica outer forearc	Sediment	36.7	8.69	0.78
**GF119S**	7954.31	2174.13	Argentina backarc	Sediment	80	7.75	0.79
**LH18F**	11274.13	3025.97	Cordillera Talamanca	Fluid	55.4	6.7	0.79
**EP17F**	3224.4	850.89	Costa Rica outer forearc	Fluid	26.4	9.99	0.79
**AO19S**	315.56	82.69	Argentina backarc	Sediment	27.8	6.25	0.79
**LC19F**	7355.95	1836.67	Argentina backarc	Fluid	84	6.94	0.8
**PG19S**	5290.06	1307.15	Argentina backarc	Sediment	43.9	8.74	0.8
**PS18S**	1165.03	286.77	Costa Rica outer forearc	Sediment	33	8.2	0.8
**PL17S**	20079.51	4798.48	Active volcanic arc	Sediment	37.6	0.85	0.81
**LE18F**	5406.9	1289.32	Cordillera Talamanca	Fluid	34.7		0.81
**BJ19S**	2057.82	474.15	Argentina backarc	Sediment	40	6.44	0.81
**LP18F**	8018.62	1783.25	Cordillera Talamanca	Fluid	39.1	6.5	0.82
**GF219F**	25.72	5.37	Argentina backarc	Fluid	80	3.21	0.83
**ER18S**	7225.99	1464.14	Active volcanic backarc	Sediment	35	9.51	0.83
**QH217F**	4871.79	978.15	Costa Rica outer forearc	Fluid	48.7	8.53	0.83
**HV221S**	142.26	28.35	Spreading center hot spot	Sediment	25.7	1.82	0.83
**MC18F**	7154.79	1377.19	Cordillera Talamanca	Fluid	31.8	9.6	0.84
**XF18F**	11532.69	2192.06	Active volcanic backarc	Fluid	28.9	7	0.84
**PG17F**	917.39	169.35	Active volcanic arc	Fluid	19.2		0.84
**LW18S**	2907.58	491.44	Costa Rica outer forearc	Sediment	31.5	7.1	0.86
**HA18F**	5644.82	936.25	Cordillera Talamanca	Fluid	33	8.9	0.86
**BR217F**	12377.42	1729.54	Active volcanic arc	Fluid	59	6.16	0.88
**PM19F**	10782.76	1501.85	Argentina backarc	Fluid	50.3	6.53	0.88
**AO19F**	399.1	54.82	Argentina backarc	Fluid	27.8	6.25	0.88
**GF119F**	12.49	1.69	Argentina backarc	Fluid	80	7.75	0.88
**BW18F**	3514.44	451.47	Cordillera Talamanca	Fluid	43.2	9.05	0.89
**BQ217F**	3.79	0.44	Active volcanic arc	Fluid	88.9	2.11	0.9
**LW18F**	14067.82	1595.78	Costa Rica outer forearc	Fluid	31.5	7.1	0.9
**QN17F**	6016.9	676.83	Active volcanic arc	Fluid	22.9	5.6	0.9
**CH18F**	4852.63	515.69	Panama slab window	Fluid	31.1	7	0.9
**CV18F**	9161.27	951.7	Panama slab window	Fluid	34.9	7.46	0.91
**CZ18F**	16531	1610.23	Panama slab window	Fluid	26.3	10	0.91
**RC18S**	5817.55	391.52	Cordillera Talamanca	Fluid	60	7.7	0.94
**PG19F**	8315.79	515.92	Argentina backarc	Fluid	43.9	8.74	0.94
**PB17S**	2689.73	112.99	Active volcanic arc	Sediment			0.96
**CI18F**	16955.03	682.48	Panama slab window	Fluid	48.3	9	0.96
**RV17F**	15451.3	576.05	Active volcanic arc	Fluid	42.7	6.19	0.96
**ES17F**	7007.89	259.52	Costa Rica outer forearc	Fluid	27.9	9.75	0.96
**KR21S**	61.39	1.58	Spreading center hot spot	Sediment	93	2.04	0.97
**SI17F**	7079.4	161.59	Costa Rica outer forearc	Fluid	35.9	9.83	0.98
**BJ19F**	3362.49	20.58	Argentina backarc	Fluid	40	6.44	0.99
**Total**	**521794.37**	**227434.98**					

### Multivariate analysis

**Fig 7:**
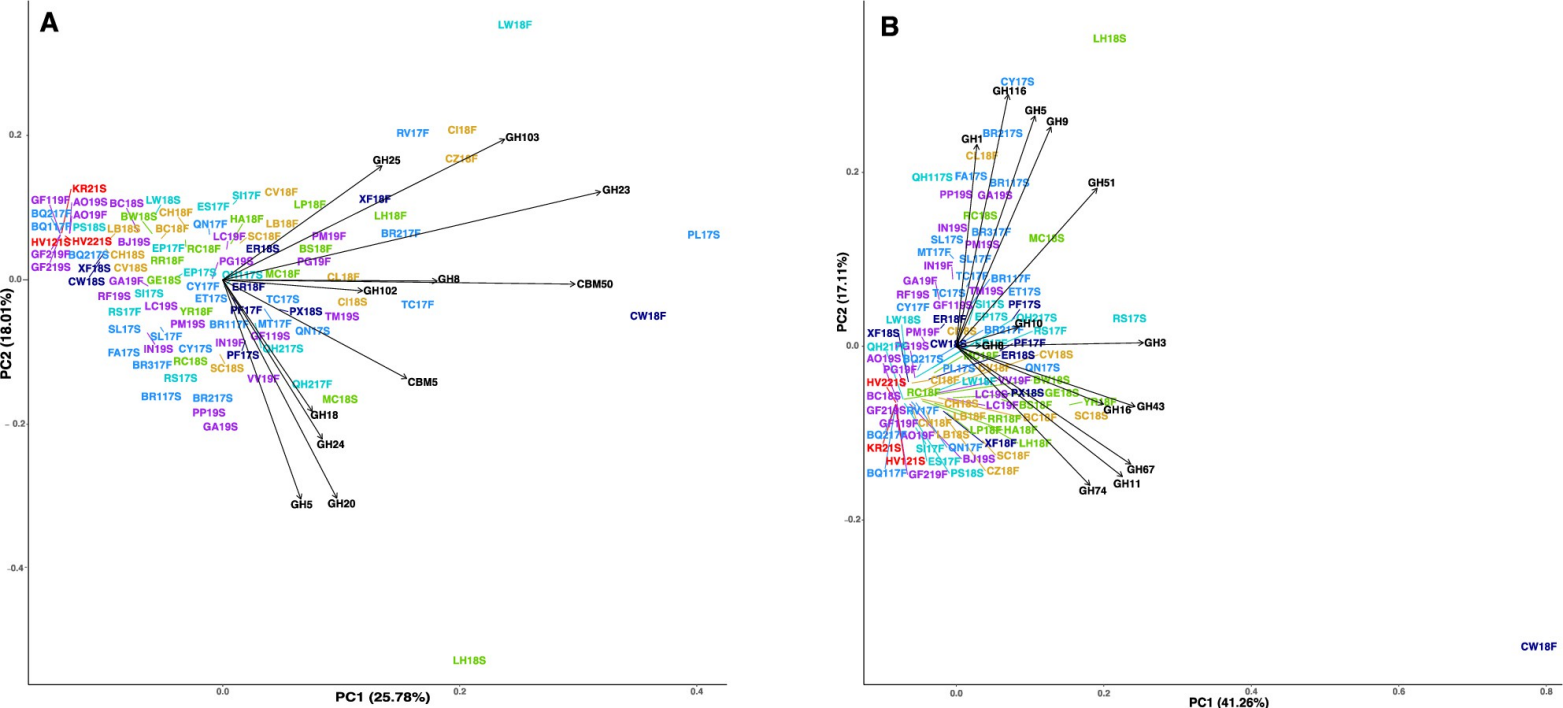
PCA plot CAZy family abundance. **A.** PCA plot of the abundance of CAZy families involved in the degradation of chitin and peptidoglycan, inferred to be cell-degrading. **B.** PCA plot of the abundance of CAZy families involved in the degradation of xylan and cellulose, inferred to be photosynthate-degrading. Sites are colored by the geological provinces shown in Fig 1.

We performed multivariate analysis on the datasets using a principal component analysis (PCA) to perform an unconstrained coordination analysis. For cell-degrading enzymes, sites do not cluster based on province (Fig 7A). Instead, we see that high temperature sites (HV121, HV221, KR21, GF119, GF219, BQ1, and BQ2) cluster together while all other sites are indistinguishable based on cell-degrading enzyme abundance. Photosynthate-degrading enzyme abundances, however, do cluster by provinces, with the Costa Rica active volcanic arc and Argentina active volcanic backarc tending to group together. Sites with volcanic activity correlate with CAZyme families GH1, GH5, GH9, GH51, and GH116, which are specifically related to cellulose degradation [58]. Here, high temperature sites continue to cluster together, but do not correlate with any specific enzymes. The nonvolcanic sites correlate with CAZy families GH16, GH43, GH74, GH11, and GH67. Families GH11, GH43, and GH67 are known to be directly related to xylan degradation [59].

To further support the idea that province separation is driven by photosynthate-degrading carbohydrate-active enzymes, rather than all peptidases, a PCA analysis was done on all the MEROPS, CAZy, and EC family annotations, as well as the annotations that correspond to the enzymes that were the target of the activity assays (S2-S5 Figs in S1 File). These PCA analyses show that there are no distinct clusters based on province. However, we see province clustering for a PCA plot of only cell-degrading and photosynthate-degrading CAZy enzymes (S6 Fig in S1 File).

## Discussion

All 63 sites produced a high diversity of enzymes predicted to be capable of breaking down organic matter outside the cell. This suggests that hot spring communities can break down a wide variety of organic compounds ranging from proteins and carbohydrates to structural molecules, as has been previously suggested [17, 61–63]. Below we will describe how the distribution of organic carbon degrading enzymes across these sites suggest these communities are primarily supported by microbial biomass, rather than plant detritus, consistent with a chemolithoauotrophically-based ecosystem. But these heterotrophic enzymes are less directly influenced by geological features than the taxonomic compositions or respiratory capabilities of these communities [64–67].

### Nature of the heterotrophic enzymes across all seeps and hot springs

We propose that the heterotrophic community in these terrestrial seeps and hot springs primarily use the organic matter of dead bacterial necromass produced *in situ* rather than allochthonous surface-derived material from plant matter. The heterotrophic community may include autotrophs that are also capable of metabolizing organic compounds. To consume complex organic matter, heterotrophs rely on extracellular enzymes such as CAZymes and peptidases to degrade the larger organic molecules so they can bring them into the cell [64]. CAZymes are important for geochemical cycling because they facilitate the breakdown of complex carbon substrates [65]. Another subset of enzymes involved in the biogeochemical cycling of heterotrophs are peptidases, which cleave peptide bonds between amino acids [60, 66]. Protein degradation has been shown to be important for heterotrophs within the subsurface [67, 68]. Heterotrophs within these springs may rely on protein from dead cells (necromass) for carbon and nitrogen acquisition, as they do in marine sediments [67, 69]. EC annotations are based on the reactions catalyzed which allows for analysis of different reactions within the assemblies, and much of the important heterotrophic enzymes fall under EC3, hydrolases [36]. The enzyme annotation methods do not distinguish between prokaryotic and eukaryotic sources [34, 36, 65]. Thus, the set of enzymes present in an environment can indicate the chemical nature, and therefore the source, of organic matter being consumed by heterotrophs [70].

An ecosystem supported by bacterial and archaeal cell necromass should contain more enzymes involved in peptidoglycan and chitin degradation such as chitinases, N-acetylglucosaminidase, and lysozymes [53–56]. Enzymes that are involved in the degradation of plant material, which in the case of subsurface springs would indicate allocthonous material from the surface, are often cellulases, xylanases, and glucosidases [57–59]. These enzymes have been characterized into CAZy families (Table 2). In our metagenomic assemblies, enzymes associated with cell necromass degradation are the most numerous overall, i.e., twice the amount of photosynthate-degrading enzyme families (Table 3). Enzymes for cell necromass consumption include those that break down peptidoglycan, a key component in bacterial cell walls. CAZyme families associated with peptidoglycan degradation are GH103, GH102, GH73, GH25, GH24, GH23, and CBM50 (Table 2) [56]. These families are found in most of the assemblies (Fig 4). The CAZyme families with the highest abundance are GH23 (peptidoglycan lyase) and CBM50 (LysM domain). These two CAZyme families are integral to the degradation of peptidoglycan.

Another facet of the heterotrophic community utilizing necromass for essential nutrients is the acquisition of starch, glycogen, and trehalose [71]. These compounds may also be derived from photosynthate materials. The CAZy families that are present within all assemblies are from the families GH13, GH15, GH31, GH57, GH122, and GH133 (Fig 4), which are involved in starch degradation [72]. This suggests that the possibility of using starches is a common heterotrophic metabolism across these sites.

The peptidase annotations also support the idea that the heterotrophic community relies on necromass for carbon and energy acquisition. Peptidase families such as S11, M23, S13, S66, M15, M74, M14, and C51 are used for peptidoglycan degradation (Fig 3). These families’ biological functions are associated with lysis or degradation of bacterial cell walls [53]. Of the eight listed cell wall degrading/lysing peptidases, all except M74 and C51 are present in every assembly. Peptidase family M74 is present in all but four of the assemblies, GF119F, GF119S, GF219F, and GF219S. Peptidase family C51 is present in all but eight of the assemblies, BQ117F, GF119F, GF119S, GF219F, GF219S, HV121S, KR21S, and RF19S. All the samples lacking M74 and C51 fall within the temperature range (80–93.5°C) in magmatic steam-heated springs. This may suggest that M74, which is a murein endopeptidase and C51, a D-alanyl-glycyl peptidase, are not adapted to high temperatures.

CAZyme families associated with plant degradation have lower total abundance in comparison to cell degradation even though there are more possibilities for plant degrading families to be found (Table 3). Of the 45 assemblies that have more than 30% plant degrading enzymes out of all the enzymes related to cell and plant degradation (Table 2), 34 are sediment samples (Table 3). Therefore, sediment assemblies contain more annotations for photosynthate-degrading enzymes than fluid samples. This may be caused by additions of photosynthetically-derived material to the sediments deposited at the surface.

The dominance of cellular biomass as the main organic matter source for the heterotrophic community is consistent with chemolithoautotrophic biomass forming the major primary production. This agrees with the findings from Barry et al., 2019a, who used helium and carbon isotopes from the same sites to show that the dissolved inorganic carbon was almost entirely derived from deep (i.e., mantle and subduction-related) sources [3]. Since hot spring enzymes related to cell necromass are more abundant in our metagenomes, we propose that hot springs microbes primarily subsist using necromass derived from chemoautotrophs rather than surficially-derived organic carbon ultimately derived from plants.

### Independent confirmation of enzyme activity with substrate proxy analyses

Extracellular enzyme assays were used as a proxy for the activity of heterotrophic organisms within the seeps and hot spring derived sediment samples. Enzymes that are commonly-assayed in soils are often associated with cellulose and lignin degradation along with enzymes that hydrolyze proteins [70]. Degradation of plant litter is often demonstrated using assays for AG, BG, CB, and XYL, as they degrade cellulose and xylan. LEU assays represent carbon and nitrogen acquisition from proteins alongside NAG which is a proxy of the degradation of peptidoglycan and chitin [73]. Extracellular enzyme assays can reveal different organic carbon and nitrogen sources for microbial communities, allowing for inferences about biogeochemical cycling within the system. Enzymes involved in carbon and nitrogen acquisition have been used to demonstrate the limitations of nutrients within various systems [70, 73, 74]. The results for these assays predicted that these organisms are not highly active (S4 Table in S1 File). However, the fact that activity could be observed at all suggests that at least some of the enzymes we identified in our metagenomic annotations were active in natural samples and the lack of extracellular enzymatic activity may be the result of not recreating the ideal geochemical parameters for the hydrolysis within the lab setting [75].

### Highest temperature magmatic steam-heated springs have low diversity of heterotrophic enzymes

Lower diversity microbial communities tend to be found at very high temperature springs [55]. Accordingly, our high temperature and low pH sites (GF219S, GF119F, GF219F, HV121S, and KR21S) have fewer annotations for both CAZymes and peptidases than more mesophilic springs. This is likely due to the small number of organisms present within these sites (S1 Fig in S1 File). Although these sites are driven by heat and volatiles originating from the subsurface, they are not representative of deep subsurface communities. The depth of the subsurface biosphere at these sites is shallow (<50m) due to the high heat flow in the area and near boiling temperature that limit the possible distribution of microorganisms at depth [11]. The shallow subsurface nature and lower residence time of the communities in these sites combined with the lack of phylogenetic diversity is the likely cause of the lower functional diversity of the heterotrophic community.

The extracellular enzymatic assays demonstrate that many high temperature sites may not be expressing the enzymes found within their metagenomes (S4 Table in S1 File). The sites with the highest extracellular activities are within the temperature range of 32.5–50°C. Extracellular enzymes require energy to produce, so organisms that are already spending energy surviving in very high temperature and low pH sites may not have excess capacity for extracellular enzyme production [67, 76, 77]. Therefore, because the hyperthermic sites are energy-limited they are not expressing as many extracellular enzymes as the more mesothermic springs.

### Heterotrophic metabolism is not influenced by geological processes

Chemolithoautotrophic metabolisms and the redox couples that provide power for them vary depending on geological province across the Central America convergent margin [4, 7]. We hypothesized that the heterotrophic community’s enzymatic functions would also vary across the provinces, since their taxonomic identities and respiratory properties do [4, 7]. However, most of the extracellular enzymes in our metagenomic assemblies do not correlate with geological provinces. Based on hierarchical clustering of spearman correlations, all sites except for the very high temperature magmatic steam-heated sites are difficult to distinguish based on abundance of predicted extracellular enzymes for peptide and carbohydrate degradation. Even though the populations of chemolithoautotrophs vary by geological setting, the biomass they produce may be similar enough that it can be broken down by similar sets of enzymes (Fig 7A), even though the heterotrophic taxa that make these enzymes also vary by geological setting [4, 7].

One group of extracellular enzymes, however, does differentiate by geological province. The PCA analysis of the photosynthate-degrading enzymes shows slight clustering of the Costa Rica active volcanic arc and backarc and the Argentina backarc (Fig 7B). These sites have the same amount of photosynthate-degrading and cell-degrading enzymes as the other provinces, but the composition of the photosynthate-degrading enzymes more closely reflects cellulose degradation with enzymes such as GH5, GH9, GH116, and GH1. Springs and seeps that lack direct magmatic influence, such as the Costa Rica outer forearc, the Cordillera Talamanca, and Panama cluster separately from the volcanic sites, with more xylan-degrading enzymes. When all CAZy families for xylan, chitin, and peptidoglycan degradation are combined, clustering based on province still occurs, suggesting a strong association of volcanic sites with cellulose-degradation and non-volcanic sites with xylan-degradation (S6 Fig in S1 File).

Since these photosynthate-degrading enzymes are more abundant in surficial sediments than in the freshly-expressed fluids, they are likely degrading surface-derived organic matter rather than chemolithoautotrophic production in the subsurface. The surface-derived substrates that are cleaved by photosynthate-degrading enzymes, however, are unlikely to come from plants, since our sites in the Costa Rica volcanic arc are in a dense jungle and our sites from the Argentina backarc that group with them are from the high altiplano desert, which is extremely dry with little vegetation. If the photosynthate-degrading enzymes were mostly driven by introduction of surrounding plants, then the Costa Rica volcanic zone should have more similar enzymes to the other Costa Rica and Panama sites, since they are close together and have similarly dense vegetation. A more likely potential source of xylan for these springs is thermophilic algae [78, 79]. Volcanic springs are often associated with higher temperatures, average 55.4°C, while the non-volcanic springs are less thermophilic with an average temperature of 37.1°C. The lower temperatures of the non-volcanic springs are closer to the optimal temperatures of growth for diverse algae, some of which are also well adapted to sulfidic and acidic sites [80]. Therefore, the non-volcanic sites may allow for algae to grow and act as a source of xylan for the heterotrophic organisms. Cellulose, which is more common in volcanic sites, is often found in cyanobacteria [81], which have a higher temperature tolerance than eukaryotic algae [82]. However, we cannot rule out the alternate possibility that these enzymes are responding to delivery of different types of subsurface-derived organic matter in the volcanic vs. non-volcanic systems.

## Conclusions

Here we present extracellular carbohydrate- and peptide-degrading enzyme potential from the metagenomes of 63 seeps and hot springs across the Central American and the Andean convergent margin (Argentinian backarc of the Central Volcanic Zone), and Iceland (mantle plume-influenced spreading center). Throughout the seven tectonic-geographic sample groups examined, we see that the heterotrophic community primarily relies on the degradation of proteins rather than carbohydrates. This is supported by the MEROPS annotations that are found in high abundance across all assemblies. The highest CAZyme and peptidase annotations are for families associated with peptidoglycan degradation. This supports the hypothesis that most of the metabolic function for heterotrophs is derived from the degradation of dead microbial cells, consistent with the major source of organic matter in this system being subsurface chemolithoautotrophic production. Very high temperature (>75°C), low pH sites (< 4), that are heated by volcanic inputs, differ from the rest of the sites based on their CAZymes and peptidases. Except for a few thermophilic peptidases, they had fewer extracellular carbon-degrading enzymes, suggesting that the secondary trophic level is less well-developed at these sites, possibly because they must put more energy into survival in these extreme conditions. Except for these high temperature sites, most extracellular CAZyme, hydrolase, and peptidase families did not differ by geological province. This suggests that, even though the taxonomic identities and respirations of the chemolithoautotrophs and heterotrophs vary by geological province [4, 7], their organic matter degrading capabilities do not. The exceptions are the photosynthate-degrading enzymes which comprised a minor component of the carbon-degrading enzymes. Volcanic sites had more cyanobacteria-degrading enzymes while non-volcanic arc sites had more algae-degrading enzymes, likely due to the difference in temperature preference of those two types of phototrophs. This study revealed that the secondary community within terrestrial geothermal systems actively participates in the carbon budget within these sites by consuming chemolithoautotrophically-derived dead cell material, with enzymatic capabilities that are independent of geological province.

## Supporting information

S1 Checklist Inclusivity in global research. (DOCX)

S1 File All supplemental tables and figures referred to in the text. (DOCX)
