# Supplementary material for: Complex organic matter degradation by secondary consumers in chemolithoautotrophy-based subsurface geothermal ecosystems
Source: PLoS One. 2023 Aug 18;18(8):e0281277. doi: 10.1371/journal.pone.0281277 (PMC10437873; doi:10.1371/journal.pone.0281277)
Supplement: S1 File — (DOCX) [file pone.0281277.s002.docx]

**Supplemental Table 1**: Metagenome assembly statistics using quast (v 4.4) on Kbase for Iceland sites.

| **Assembly** | **HV121S** | **HV221S** | **KR21S** |
| --- | --- | --- | --- |
| # contigs (>= 0 bp) | 912 | 4344 | 3214 |
| # contigs (>= 1000 bp) | 911 | 4343 | 3213 |
| # contigs (>= 5000 bp) | 206 | 107 | 461 |
| # contigs (>= 10000 bp) | 101 | 10 | 219 |
| # contigs (>= 25000 bp) | 32 | 1 | 74 |
| # contigs (>= 50000 bp) | 15 | 1 | 17 |
| Total length (>= 0 bp) | 5645364 | 7802221 | 12561178 |
| Total length (>= 1000 bp) | 5644938 | 7801839 | 12560656 |
| Total length (>= 5000 bp) | 4095358 | 847764 | 7120134 |
| Total length (>= 10000 bp) | 3358147 | 207792 | 5442995 |
| Total length (>= 25000 bp) | 2336396 | 83915 | 3197349 |
| Total length (>= 50000 bp) | 1771546 | 83915 | 1200955 |
| # contigs | 911 | 4343 | 3214 |
| Largest contig | 230187 | 83915 | 117567 |
| Total length | 5644938 | 7801839 | 12561178 |
| GC (%) | 36.1 | 38.52 | 37.45 |
| N50 | 15540 | 1784 | 7154 |
| N70 | 4386 | 1290 | 2285 |
| L50 | 58 | 1331 | 319 |
| L75 | 236 | 2601 | 1181 |
| # N's per 100 kbp | 122.06 | 32.04 | 107.31 |

**Supplemental Table 2**: Metagenome assemblies from 2018 and 2019 datasets from Costa Rica, Panama, and Argentina

| **Assembly** | **No. Contigs** | **Total Size (B)** |
| --- | --- | --- |
| **AO19F** | 8121 | 58496569 |
| **AO19S** | 6150 | 25931833 |
| **AR17S** | 1343 | 3677960 |
| **BC18F** | 70603 | 2.12E+08 |
| **BC18S** | 1921 | 12687778 |
| **BJ19S** | 5931 | 47197600 |
| **BQ117F** | 4847 | 12976536 |
| **BQ217F** | 1588 | 9664098 |
| **BQ217S** | 881 | 5219031 |
| **BR317S** | 3103 | 52622795 |
| **BR117F** | 14010 | 1.25E+08 |
| **BR217F** | 15625 | 1.04E+08 |
| **BR117S** | 18542 | 1.7E+08 |
| **BR217S** | 48221 | 3.61E+08 |
| **BS18F** | 150037 | 3.79E+08 |
| **BW18S** | 136180 | 385362999 |
| **CH18F** | 119398 | 3.1E+08 |
| **CH18S** | 125756 | 289334943 |
| **CI18F** | 43018 | 1.55E+08 |
| **CI18S** | 24781 | 145014266 |
| **CL18F** | 81587 | 3.43E+08 |
| **CV18F** | 69989 | 246018183 |
| **CV18S** | 97434 | 276002127 |
| **CW18F** | 37673 | 1.74E+08 |
| **CW18S** | 138318 | 321497305 |
| **CY17F** | 9532 | 48220140 |
| **CY17S** | 3356 | 49712012 |
| **CZ18F** | 72813 | 228655289 |
| **EP17F** | 6997 | 39537221 |
| **EP17S** | 11330 | 85620639 |
| **ER18F** | 47911 | 1.28E+08 |
| **ER18S** | 61880 | 1.86E+08 |
| **ES17F** | 344 | 64866979 |
| **ET17S** | 25029 | 1.51E+08 |
| **FA17S** | 6453 | 87853872 |
| **GA19F** | 2811 | 17903404 |
| **GA19S** | 3929 | 33351531 |
| **GE18S** | 150572 | 3.75E+08 |
| **GF119F** | 563 | 9621853 |
| **GF119S** | 2569 | 26031841 |
| **GF219F** | 488 | 10679524 |
| **GF219S** | 569 | 8678655 |
| **HA18F** | 54319 | 2.32E+08 |
| **HV121S** | 912 | 5645364 |
| **HV221S** | 4344 | 7802221 |
| **IN19F** | 1951 | 11159695 |
| **IN19S** | 22685 | 1.19E+08 |
| **KR21S** | 3214 | 12561178 |
| **LB18F** | 83132 | 2.27E+08 |
| **LB18S** | 77944 | 1.66E+08 |
| **LC19F** | 1115 | 12558028 |
| **LC19S** | 1814 | 12445910 |
| **LE18F** | 164798 | 5.09E+08 |
| **LE18S** | 134586 | 3.45E+08 |
| **LH18F** | 92911 | 3.29E+08 |
| **LH18S** | 78751 | 3.13E+08 |
| **LP18F** | 118997 | 3.72E+08 |
| **LW18F** | 6318 | 43534387 |
| **LW18S** | 98994 | 2.32E+08 |
| **MC18F** | 61879 | 1.51E+08 |
| **MC18S** | 79033 | 3.04E+08 |
| **MT17F** | 23 | 5736255 |
| **PB17S** | 4300 | 22429571 |
| **PF17F** | 3744 | 26222332 |
| **PF17S** | 13278 | 77619353 |
| **PG17F** | 2764 | 15055679 |
| **PG19F** | 8783 | 39170972 |
| **PG19S** | 6521 | 38928961 |
| **PG17S** | 58939 | 3.76E+08 |
| **PL17S** | 6914 | 49565790 |
| **PM19F** | 7814 | 53460399 |
| **PM19S** | 10375 | 58355891 |
| **PP19S** | 8169 | 55125847 |
| **PS18S** | 44051 | 74662476 |
| **PX18S** | 143045 | 4.2E+08 |
| **QH217F** | 2839 | 32772539 |
| **QH117S** | 4209 | 86093826 |
| **QH217S** | 14370 | 1.11E+08 |
| **QN17F** | 2350 | 15784361 |
| **QN17S** | 68636 | 2.37E+08 |
| **RC18F** | 45514 | 1.29E+08 |
| **RC18S** | 101361 | 3.48E+08 |
| **RF19S** | 3658 | 30313326 |
| **RR18F** | 101302 | 2.47E+08 |
| **RS17F** | 3553 | 18643462 |
| **RS17S** | 25272 | 1.63E+08 |
| **RV17F** | 8302 | 50313599 |
| **SC18F** | 72223 | 2.67E+08 |
| **SC18S** | 71898 | 2.79E+08 |
| **SI17F** | 878 | 8670679 |
| **SI17S** | 20157 | 2.23E+08 |
| **SL17F** | 1481 | 11833810 |
| **SL17S** | 16272 | 95296180 |
| **TC17F** | 1996 | 16728578 |
| **TC17S** | 10638 | 60705063 |
| **TM19S** | 1170 | 10685610 |
| **VV19F** | 8144 | 45629789 |
| **XF18F** | 90274 | 2.93E+08 |
| **XF18S** | 109171 | 2.42E+08 |
| **YR18F** | 130198 | 3.51E+08 |

**Supplementary Table 3:** Geochemical values for DIC, DOC, Nitrite, Phosphate, Sulfate, TIC, TN, and TOC. Empty cells show that these measurements were not made for these samples.

| **Abb.** | **DO** | **Sal** | **DIC** | **DOC** | **ðC13** | **ðC12** | **TOC** | **TN** | **so4** | **no3** | **po4** | **nh4** |
| --- | --- | --- | --- | --- | --- | --- | --- | --- | --- | --- | --- | --- |
| AO19 | 90 | 21.21 | 5.45 |  | -7.66 |  |  |  |  |  |  |  |
| BC18 | 51 | 23.87 | 56.85 |  | 2.34 |  |  |  | 0 |  |  |  |
| BJ19 | 10 | 8.65 | 15.92 |  |  |  |  |  |  |  |  |  |
| BQ17 | 9.9 | 5.73 |  |  |  |  | 0.1 | 1.29 | 25 | 0.02 | 0 | 4.76 |
| BR117 | 41 | 7.02 | 9.56 | 0.8 | 0.23 | -11.16 | 1.65 | 0.02 | 7.76 | 0 | 0 | 0 |
| BR217 | 7 | 6.71 | 9.31 | 0.89 | 0.96 | -10.75 | 1.24 | 0.15 | 7.41 | 0 | 0 | 0 |
| BS18 | 20 | 3.1 |  |  |  |  | 0.31 |  | 0 |  |  |  |
| BW18 | 3 | 3.13 | 0.36 |  | -14.14 |  |  |  | 0 |  |  | 0.48 |
| CH18 | 41 | 15.3 | 37.97 |  | 2.01 |  | 12.93 |  | 17.72 |  |  |  |
| CI18 | 18 | 1.11 | 0.12 |  | -14.23 |  | - |  | 2.41 |  |  | 0.32 |
| CL18 | 29 | 3.26 | 0.74 |  | -5.29 |  | 0.14 |  | 11.78 |  |  |  |
| CV18 | 14 | 3.75 | 0.89 |  | -1.14 |  |  |  | 0 |  |  | 0.76 |
| CW18 |  |  |  |  |  |  | 14.8 |  |  |  |  | 0.45 |
| CY17 | 26.7 | 6.8 | 5.24 | 1.82 | -4.99 | -7.78 | 0.25 | 0.04 |  | 0.04 | 0 | 0.09 |
| CZ18 | 11 | 0.22 | 0.86 |  | -13.62 |  | 0.64 |  | 0 |  |  | 0.08 |
| EP17 | 4 | 0.17 | 0.55 | 0.22 | -21.57 | -25.48 | 3.1 | 0.06 | 0 | 0.05 | 0 | 0 |
| ES17 | 48.3 | 0.2 | 1.14 |  | -17.98 | -24.18 |  |  | 0 | 0.08 | 0 | 0.59 |
| ER18 |  |  |  |  |  |  | 1.39 |  |  |  |  | 0.15 |
| ET17 | 13.4 | 2.02 |  |  |  |  | 1.05 | 0.16 | 2.38 | 0.05 | 0 | 0 |
| FA17 | 10.3 | 6.39 |  |  |  |  | 1.23 | 0.19 | 9.39 | 0.03 | 0 | 3.57 |
| GA19 | 10 | 3.28 | 6.26 |  | -1.5 |  |  |  |  |  |  |  |
| GE18 | 85 | 0.45 | 1.22 | 1.19 | -18.15 | -28.42 | 0.48 |  | 0.61 |  |  | 21.45 |
| GF119 | 10 | 0.19 | 0.34 |  | -14.81 |  |  |  |  |  |  |  |
| GF219 | 60 | 1.02 | 0.48 |  | -12.4 |  |  |  |  |  |  |  |
| HA18 | 26 | 4.97 | 0.12 |  | -21.19 |  |  |  | 8.46 |  |  | 0.25 |
| HV121 | 98 | 0.93 |  |  |  |  |  |  |  |  |  |  |
| HV221 | 143 | 1.09 |  |  |  |  |  |  |  |  |  |  |
| IN19 | 10 | 1.54 |  |  |  |  |  |  |  |  |  |  |
| KR21 | 100 | 1.23 |  |  |  |  |  |  |  |  |  |  |
| LB18 | 70 | 25.35 | 17.74 |  | 3.55 |  | 1.17 |  | 0 |  |  |  |
| LC19 | 20 | 6.1 | 6.82 |  | -2.62 |  |  |  |  |  |  |  |
| LE18 |  |  |  |  |  |  | 0.45 |  |  |  |  | 0.27 |
| LH18 | 12 | 8.43 | 33.54 |  | -2.2 |  | 0.42 |  | 2.9 |  |  |  |
| LP18 | 32 | 5.88 | 31.33 |  | -1.47 |  | 3.49 |  | 1.74 |  |  | 0.18 |
| LW18 | 3.5 | 0.55 | 7.11 |  | -2.95 |  | 0.53 |  |  |  |  | 0.17 |
| MC18 | 23 | 2.33 | 0.04 | 0.44 | -14.31 | -26.86 | 0.28 |  | 3.01 |  |  |  |
| MT17 | 12.4 | 7.21 | 21.7 | 1.66 | 3.55 | -7.38 | 0.25 | 0.02 | 0.56 | 0 | 0 | 0 |
| PB17 |  |  |  |  |  |  |  |  |  |  |  |  |
| PF17 | 4.3 | 4.18 | 23.72 | 1.16 | 1.24 | -5.15 | 1.66 | 0.3 | 0.57 | 0 | 0 | 0 |
| PG19 | 50 | 0.61 | 0.35 |  | -15.33 |  |  |  |  |  |  |  |
| PG17 |  |  |  |  |  |  | 0.15 | 0.04 | 0 | 0 | 0 | 0.01 |
| PL17 | 46.6 | 136.68 | 0.86 | 0.57 | -2.97 | -20.66 | 0.02 |  | 25 | 0 | 0 | 0.02 |
| PM19 | 10 | 5.09 | 21.14 |  | -5.33 |  |  |  |  |  |  |  |
| PP19 | 0 | 1.81 | 2.15 |  | -10.67 |  |  |  |  |  |  |  |
| PS18 | 122 | 69.2 | 3.18 |  | -9.32 | -25.21 | 0.18 |  | 15.61 |  |  |  |
| PX18 |  |  |  | 1.21 |  |  | 0.67 |  |  |  |  | 0.12 |
| QH117 | 2.5 | 4.64 | 0.1 |  | -9.93 | -27.58 | 7.13 | 3.23 | 4.3 | 0.1 | 0 | 2.35 |
| QH217 | 47.3 | 2.94 | 0.11 | 0.17 | -17.98 | -22.37 | 0.96 | 0.85 | 2.05 | 0.05 | 0 | 0.05 |
| QN17 | 84 | 0.17 | 2.81 | 0.22 | -1.95 | -13.82 | 0.21 | 0.03 | 0 | 0.04 | 0 | 0.52 |
| RC18 | 45 | 2.83 | 1.07 | 0.42 | -5.37 | -22.55 | 7.57 |  | 2.79 |  |  | 0.25 |
| RF19 | 0 | 1.57 | 3.45 | 0.53 | -8.28 |  |  |  |  |  |  |  |
| RR18 | 73 | 2.51 | 0.32 |  | -18.14 |  | 0.08 |  | 5.97 |  |  | 0.14 |
| RS17 | 5.6 | 0.24 | 0.25 |  | -16.04 | -24.55 | 1.19 | 0.1 | 0 | 0 | 0 | 0.01 |
| RV17 | 1.8 | 62.86 | 19.73 | 0.3 | -0.28 | -4.71 |  |  | 6.56 | 0 | 0 | 0 |
| SC18 | 30 | 56.21 | 58.45 | 2.87 | 3.34 |  | 7.92 |  | 0 |  |  |  |
| SI17 | 36.2 | 3.87 | 2.24 |  | -6.43 | -18.27 | 4.45 | 0.35 | 0 | 0.03 | 0 | 0 |
| SL17 | 22 | 3.09 | 5.69 | 0.4 | -5.28 | -0.65 | 3.15 | 0.24 | 7.2 | 0.03 | 0 | 0.31 |
| TC17 | 18.7 | 3.91 | 13.57 | 6.29 | -2.36 | -7.94 | 5.04 | 0.92 | 2.38 | 0.05 | 0 | 1.33 |
| TM19 | 40 | 3.93 | 19.03 |  | -7.18 |  |  |  |  |  |  |  |
| VV19 | 60 | 0.4 | 0.66 |  | -13.28 |  |  |  |  |  |  |  |
| XF18 |  |  |  |  |  |  | 1.86 |  |  |  |  | 0.2 |
| YR18 | 10 | 4.59 | 0.06 |  | -21.07 |  |  |  | 0 |  |  |  |

**Supplemental Table 4:** Enzymatic activities per site sediment. Activities are represented as (µmol/g/hour). Negative values are assumed to be no activity. Activities in bold are values that are greater than 1 µmol/g/hour. Enzymes abbreviations are alpha-glucosidase (AG), beta-glucosidase (BG), cellobiohydrolase (CB), leucine aminopeptidase (LEU), N-acetyl-β-D-glucosaminidase (NAG), phosphatase (PHOS), sulfatase (SULF), and xylosidase (XYL).

| ***Abb.*** | ***Temp °C*** | ***AG*** | ***BG*** | | ***CB*** | | ***LEU*** | ***NAG*** | ***PHOS*** | ***SULF*** | ***XYL*** |
| --- | --- | --- | --- | --- | --- | --- | --- | --- | --- | --- | --- |
| *AO19S* | 30 | 0.03 | 0.03 | 0.03 | | 0.01 | | 0.03 | 0.02 | 0.03 | 0.03 |
| *BJ19S* | 40 | -0.14 | -0.18 | -0.13 | | 0 | | 0.31 | 0 | 0.1 | -0.14 |
| *BQ2117S* | 70 | 0.01 | 0.01 | 0.04 | | -0.01 | | 0 | 0 | 0.01 | 0.01 |
| *BQ117S* | 70 | 0 | 0.04 | 0.07 | | -0.01 | | 0.01 | 0 | 0.01 | 0.04 |
| *BR117S* | 55 | -0.2 | -0.04 | -0.18 | | 0.01 | | 0.12 | 0.02 | 0.08 | -0.18 |
| *BS18S* | 50 | 0.68 | **2.1** | **1.89** | | -0.01 | | 0.18 | 0.01 | -0.29 | **1.94** |
| *CH18S* | 32.5 | **2.19** | **9.04** | **5.43** | | -0.74 | | **11.6** | **1.05** | **3.14** | **12.54** |
| *CL18S* | 50 | -0.26 | 0.34 | 0.61 | | -0.02 | | 0.16 | -0.05 | -0.24 | 0.49 |
| *CV18S* | 32.5 | 0.2 | 0.17 | 0.18 | | 0.13 | | 0.14 | 0.03 | 0.08 | 0.16 |
| *CW18S* | 32.5 | 0.89 | 0.34 | **1.12** | | 0.06 | | 0.46 | 0.05 | **1.23** | 0.51 |
| *CY17S* | 70 | 0.14 | 0.1 | 0.11 | | 0.01 | | 0.02 | 0 | 0.11 | 0.1 |
| *CZ18S* | 32.5 | -2.9 | **3.21** | -7.5 | | -0.02 | | 0.96 | 0.12 | -0.2 | -2.75 |
| *EP17S* | 30 | -0.04 | -0.04 | -0.04 | | 0.02 | | -0.04 | 0 | -0.04 | -0.04 |
| *ET17S* | 40 | -0.09 | 0.03 | -0.09 | | 0 | | -0.12 | 0 | 0.24 | -0.11 |
| *FA17S* | 55 | 0.01 | 0.03 | 0.01 | | 0 | | 0.03 | 0.01 | 0.02 | 0.02 |
| *GA19S* | 70 | 0.01 | 0.02 | 0.01 | | 0 | | 0.01 | 0.01 | -0.03 | 0 |
| *GE18S* | 32.5 | 0.48 | 0.4 | 0.52 | | 0.04 | | 0.39 | 0.07 | 0.58 | 0.48 |
| *GF119S* | 70 | 0.69 | 0.11 | -0.6 | | 0 | | 0.01 | 0.01 | -0.16 | 0.72 |
| *GF219S* | 70 | -0.07 | 0.05 | 0.3 | | -0.03 | | 0.01 | 0 | 0.02 | 0.08 |
| *HV121S* | 70 | -0.03 | -0.03 | -0.03 | | -0.12 | | 0.04 | 0 | -0.03 | -0.01 |
| *HV221S* | 70 | -0.03 | 0 | -0.02 | | -0.06 | | 0.03 | 0 | -0.03 | 0 |
| *IN19S* | 40 | -0.1 | -0.11 | -0.1 | | 0.01 | | -0.16 | 0.01 | 0 | -0.09 |
| *KR21S* | 70 | -0.05 | -0.07 | -0.07 | | 0.01 | | 0.03 | 0 | -0.05 | -0.03 |
| *LB18S* | 32.5 | 0.56 | 0.28 | 0.64 | | 0 | | 0.32 | 0.04 | 0.06 | 0.47 |
| *LC19S* | 70 | -0.36 | -0.5 | -0.41 | | -0.05 | | 0.01 | 0.02 | -0.2 | -0.29 |
| *LE18S* | 32.5 | 0.17 | 0.24 | 0.2 | | 0.38 | | 0.27 | 0.1 | 0.04 | 0.28 |
| *LH18S* | 50 | 0.48 | 0.19 | 0.27 | | 0.01 | | 0.12 | 0.22 | -0.13 | 0.39 |
| *LP18S* | 50 | -1.01 | **0.9** | **0.96** | | -0.02 | | 0.15 | 0.06 | 0.04 | **1.66** |
| *LW18S* | 32.5 | -0.18 | -0.2 | -0.23 | | -0.02 | | -0.22 | 0.02 | -0.27 | -0.15 |
| *MC18S* | 32.5 | -0.63 | -0.61 | -0.77 | | -0.04 | | -0.69 | 0.31 | 0.01 | -0.25 |
| *MT17S* | 55 | -2.97 | **2.97** | -5.6 | | 0.03 | | 0.23 | 0.13 | -0.85 | **3.85** |
| *PF17S* | 30 | 0 | 0.02 | 0 | | 0 | | 0.04 | 0 | 0.02 | 0.01 |
| *PG17S* | 40 | **1.38** | 0.2 | **0.96** | | 0.18 | | 0.15 | 0 | 0.02 | 0.39 |
| *PG19S* | 40 | -0.12 | 0.13 | -0.1 | | 0.01 | | 0.25 | 0.02 | 0.31 | -0.06 |
| *PL17S* | 40 | -0.03 | -0.04 | -0.05 | | -0.09 | | -0.03 | 0.02 | 0.04 | -0.04 |
| *PM19S* | 55 | 0.84 | 0.86 | **2.67** | | 0.01 | | 0.13 | 0.03 | 0.5 | **2.17** |
| *PS18S* | 32.5 | 0.31 | 0.56 | 0.46 | | 0.15 | | 0.55 | 0.05 | 0.21 | -0.2 |
| *PX18S* | 32.5 | -0.77 | -17.78 | **10.61** | | -0.17 | | **18.83** | 0.35 | **6.08** | -31.15 |
| *QH117S* | 40 | -0.14 | -0.15 | -0.14 | | 0 | | -0.03 | 0.01 | -0.79 | -0.14 |
| *QH217S* | 40 | -0.04 | -0.04 | -0.04 | | 0.01 | | -0.04 | 0 | -0.08 | -0.04 |
| *QN17S* | 30 | 0.02 | -0.01 | -0.09 | | 0.01 | | 0 | 0.03 | 0.01 | 0.09 |
| *RC18S* | 50 | 0.06 | 0.05 | -0.57 | | 0 | | 0.05 | 0 | 0.1 | 0.39 |
| *RR18S* | 50 | 0.55 | 0.23 | 0.31 | | 0.01 | | 0.21 | 0.02 | -0.22 | -0.84 |
| *RS17S* | 30 | 0.04 | 0.17 | 0.03 | | -0.01 | | 0.13 | 0.03 | 0.6 | 0.06 |
| *RV17S* | 40 | 0.52 | 0.27 | **1.42** | | 0.24 | | 0.16 | 0.01 | 0.14 | 0.82 |
| *SL17S* | 55 | -0.34 | -0.25 | -0.31 | | 0.03 | | 0.19 | 0.06 | -0.23 | -0.28 |
| *SI17S* | 40 | -2.56 | -3.27 | 0.73 | | -0.27 | | 0.49 | 0.03 | 0.18 | **1.49** |
| *TC17S* | 70 | -0.06 | -0.07 | -0.08 | | 0.01 | | 0.01 | 0.01 | -0.06 | -0.08 |
| *TM19S* | 70 | -0.12 | -0.11 | -0.12 | | -0.02 | | 0.01 | 0.01 | -0.12 | -0.11 |
| *XF18S* | 32.5 | -0.19 | -0.17 | -0.18 | | -0.02 | | -0.09 | 0.02 | 0.09 | -0.16 |

**Supplemental Table 5**: Ttest significance table for enzymes degrading photosynthates vs. cells. Ttest calculated based on abundance of each enzyme within sediments and fluids per site.

| Enzyme | Photosynthate | Cell | Significance |
| --- | --- | --- | --- |
| GH116 | + | - | 0.00033315 |
| GH103 | - | + | 0.00039493 |
| GH20 | - | + | 0.00047599 |
| GH5 | + | - | 0.00135728 |
| GH23 | - | + | 0.00240815 |
| GH9 | + | - | 0.00648625 |
| GH51 | + | - | 0.00986168 |
| GH102 | - | + | 0.01933883 |
| GH25 | - | + | 0.07429471 |
| CBM5 | - | + | 0.08289922 |
| GH3 | + | - | 0.13637659 |
| GH8 | + | - | 0.1375164 |
| GH24 | - | + | 0.17638535 |
| GH16 | + | - | 0.18207355 |
| GH18 | - | + | 0.20921345 |
| CBM50 | - | + | 0.23303818 |
| GH43 | + | - | 0.25413116 |
| GH67 | + | - | 0.31224325 |
| GH10 | + | - | 0.36150091 |
| GH11 | + | - | 0.37448346 |
| GH74 | + | - | 0.49078569 |

**Supplemental Figure 1**: **Taxonomy for Iceland reads.** Taxonomic identification was found using Gottcha2 on Kbase. The percent relative abundance of the reads in each site are shown on the y-axis. The genus level taxonomy is displayed on the x-axis.

**Supplemental Figure 2**: **MEROPs family abundance PCA plot.**

**Supplemental Figure 3**: **EC hydrolase abundance PCA plot.**

**Supplemental Figure 4:** **CAZy family abundance PCA plot.**

**Supplemental Figure 5**: **The read abundance metagenome annotations of enzymes used in assay (AG, BG, CB, NAG, LEU, PHOS, SULF, XYLS) PCA without any geochemical parameters.**

**Supplemental Figure 6**: **Degradation PCA plot.** PCA analysis of all cell and photosynthate degrading CAZy family abundances per assembly.
